# Supplementary material for: Impact of physicians’ participation in non-interventional post-marketing studies on their prescription habits: A retrospective 2-armed cohort study in Germany
Source: PLoS Med. 2020 Jun 26;17(6):e1003151. doi: 10.1371/journal.pmed.1003151 (PMC7319278; doi:10.1371/journal.pmed.1003151)
Supplement: S1 Appendix — (DOCX) [file pmed.1003151.s001.docx]

**S1 Appendix. Selection of alternative drugs**

Alternative drugs were selected by identifying all drugs that were identical with the studied drug in the non-interventional post-marketing studies (NIPMSs) regarding the ATC-code until the 3^rd^ place. Of these drugs, those were selected that were marketed for the same therapeutic indication as noted in the summary of product information. For example, for the NIPMS studying Fluocinolon acetonide, with the ATC-code S01BA15, all drugs with an ATC-code beginning with S01B were acquired, but only those that were used for the same therapeutic indication as the studied drug (in this case, diabetic macular edema) were used as comparable drugs for matching as well as analyses regarding prescription shift.
