# Supplementary material for: Impact of physicians’ participation in non-interventional post-marketing studies on their prescription habits: A retrospective 2-armed cohort study in Germany
Source: PLoS Med. 2020 Jun 26;17(6):e1003151. doi: 10.1371/journal.pmed.1003151 (PMC7319278; doi:10.1371/journal.pmed.1003151)
Supplement: S2 Appendix — (DOCX) [file pmed.1003151.s002.docx]

**S2 Appendix. Matching Methods**

For the matching procedure, only physicians with continuous prescriptions in every time period t0, t1 and t2 were included (i.e. on average a minimum of 50 prescribed packages per month of any drug). For every LANR (a unique ID number permanently assigned to each physician) belonging to a physician taking part in an NIPMS, two control-LANR were selected with a similar prescription behavior during t0 based on their Mahalanobis distance via R optmatch package (Version: 0.9-8, R environment for statistical computing version 3.4.3). Specifically, three criteria were used to assess prescription behavior and factored equally into matching: overall number of prescribed packages, number of prescribed defined daily doses (DDD) of the studied drug and number of DDD of the comparable drugs. Each LANR was used only once as a control over all NIPMSs, i.e. controls were drawn without replacement.
