# Supplementary material for: Impact of physicians’ participation in non-interventional post-marketing studies on their prescription habits: A retrospective 2-armed cohort study in Germany
Source: PLoS Med. 2020 Jun 26;17(6):e1003151. doi: 10.1371/journal.pmed.1003151 (PMC7319278; doi:10.1371/journal.pmed.1003151)
Supplement: S3 Appendix — (DOCX) [file pmed.1003151.s003.docx]

**S3 Appendix. Validation Process**

To ensure the accuracy of the LANR from the NIPMS notifications and because of the occasional absence of a LANR, a lookup- and validation process was conducted by comparing the acquired NIPMS data with the official Statutory Health Insurance (SHI)-practitioners directory. Due to data protection limitations for the use of the SHI-directory the process took place at a temporally prepared secured workspace at IKK Southwest insurance in Mainz between Jan 22^nd^ and 26^th^ 2018.

To improve the matching rate between the original information of the NIPMS-physicians and the SHI-physician directory, both lists were normalized before the lookup and validation process. This included changing characters to lower case, deletion of special characters and stopwords, and standardization of office address formats. The lookup and validation routines ran semiautomatically in an iterated loop of qualified combinations of available features like surname, name, zip codes, town, street, house number and unique office ID and the final elimination of duplicates. The original list was thus reduced from 5,513 entries to a list of 5,065 validated entries consisting of combinations of NIPMS reference and LANR. This list was returned to the National Association of Statutory Health Insurance Funds for the extraction of the research dataset.
