# Supplementary material for: Impact of physicians’ participation in non-interventional post-marketing studies on their prescription habits: A retrospective 2-armed cohort study in Germany
Source: PLoS Med. 2020 Jun 26;17(6):e1003151. doi: 10.1371/journal.pmed.1003151 (PMC7319278; doi:10.1371/journal.pmed.1003151)
Supplement: S4 Appendix — (DOCX) [file pmed.1003151.s004.docx]

**S4 Appendix. Marketing Indicators**

Marketing indicators were defined as characteristics of NIPMSs that suggest that the NIPMS was initiated for marketing purposes. We chose these characteristics knowing that for each of them, there may be other, non-marketing-related reasons, so that each indicator in and of itself is not enough to suggest a marketing purpose of the NIPMS. However, when several indicators are present, this may be a sign that the marketing purpose of the NIPMS carries more weight than the scientific objective. We calculated a marketing index by adding the points for each of the indicators present. The indicators were weighted by importance (see the number of points in the list below for each indicator). The indicators we chose were the following:

- *Remuneration is inappropriate or not clearly warranted:* This seemed to be the most important indicator of a marketing-purpose, as an inappropriately high remuneration suggests that the remuneration is not only compensation for the physician’s effort but additionally an incentive to prescribe the studied drug. For evaluation of appropriateness, a minimum amount of remuneration was set (€250 per patient, €100 per physician-patient-interaction on average, or €150 for a single patient-physician interaction) below which all remuneration was considered appropriate. A remuneration of €75 per hour was assumed as appropriate following Ruppert et al. [1].The appropriateness was then rated independently by two raters and where the two raters differed, a consensus was reached after a discussion. A remuneration above the mentioned €75 per hour was considered inappropriate (2 pts. on the marketing index). When there was insufficient information to determine appropriateness, the remuneration was rated as insufficiently justified (1 pt. on the marketing index). All other remuneration was considered appropriate (0 pts. on the marketing index).
- *Negligible effort*: When the physicians’ role in the NIPMS was negligible, i.e. the work could also be completed by someone of another occupation or the effort was not likely to result in a relevant contribution to the study, this was counted as a marketing indicator. Two raters rated independently whether the amount of time required of the physician was less than 15 min. and as above, where the two raters differed, a consensus was reached after a discussion (0.5 pts. on the marketing index).
- *Low scientific/formal quality:* A low scientific and formal quality is another indicator for marketing use because if the scientific objective were the primary goal, the NIPMS would have to be scientifically sound and well documented. Both indicators were rated as present when less than four of six indicators of good scientific or formal quality were present (2 pts. for scientific and 1 pt. for formal quality, respectively). The indicators of good scientific and formal quality were based on recommendations by the German authorities for NIPMSs and previous studies on the topic. For scientific quality, they included: definition of a reasonable study objective, definition of reasonable end points, description of measures taken to control confounders, calculation of a sample size, design as a prospective cohort study, definition of inclusion and exclusion criteria. For formal quality, they included: description of case report form, description of quality management, description of statistic analysis, responsibilities for quality management specified, consultation with an ethics committee, publication planned.
- *Drug has been on the market for too long*: This characteristic was chosen because for drugs that have been marketed for a long time, no new information is to be expected from the initiation of an NIPMS. Based on previous estimates by other authors, we set this duration at 10 years for trademark drugs and 5 years for generics (0.5 pts on the marketing index) [2].
- *Required report missing*: The lack of a plan for publication of results was also seen as an indicator for marketing use. This indicator was rated as present when publication was not mentioned as being planned or there was no report of the data on the site of the relevant authority within 12 months after conclusion of the study (0.5 pts. on the marketing index).
- *Secrecy clause*: If the sample contracts between sponsors of the NIPMS and physicians contained a secrecy clause, this was considered suspicious because of the resulting lack of transparency and the risk that adverse events may not be reported to the authorities (0.5 pts. on the marketing index) [3].

1. Ruppert T, Hahn M, Hundt F. Remuneration for non-interventional studies – results of a survey in the pharmaceutical industry in Germany. GMS Ger Med Sci [Internet]. 2012 Feb 7 [cited 2017 Nov 1];10. Available from: https://www.ncbi.nlm.nih.gov/pmc/articles/PMC3278977/

2. von Jeinsen BKJG, Sudhop T. A 1-year cross-sectional analysis of non-interventional post-marketing study protocols submitted to the German Federal Institute for Drugs and Medical Devices (BfArM). Eur J Clin Pharmacol. 2013 Jul;69(7):1453–66.

3. Spelsberg A, Prugger C, Doshi P, Ostrowski K, Witte T, Hüsgen D, et al. Contribution of industry funded post-marketing studies to drug safety: survey of notifications submitted to regulatory agencies. BMJ. 2017 Feb 7;356:j337.
