# Supplementary figures and images for: Impact of physicians’ participation in non-interventional post-marketing studies on their prescription habits: A retrospective 2-armed cohort study in Germany
Source: PLoS Med. 2020 Jun 26;17(6):e1003151. doi: 10.1371/journal.pmed.1003151 (PMC7319278; doi:10.1371/journal.pmed.1003151)

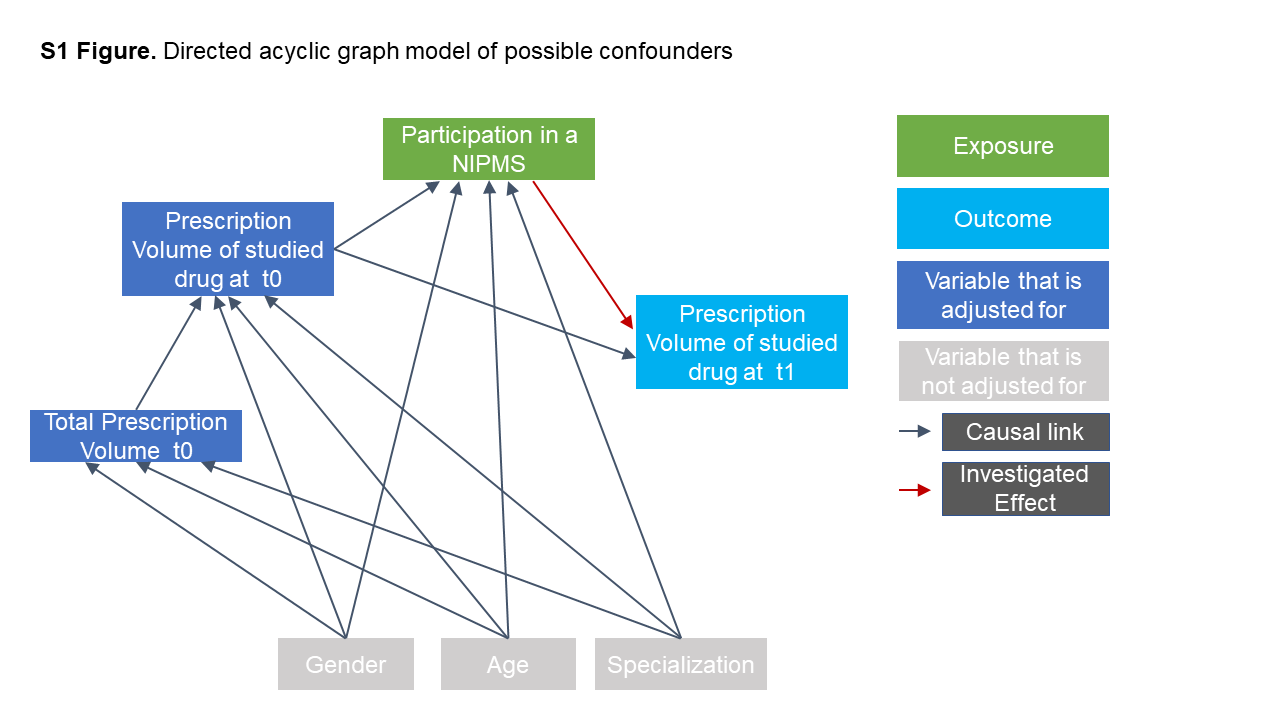

Supplement: S1 Fig — (TIF) [file pmed.1003151.s005.tif]

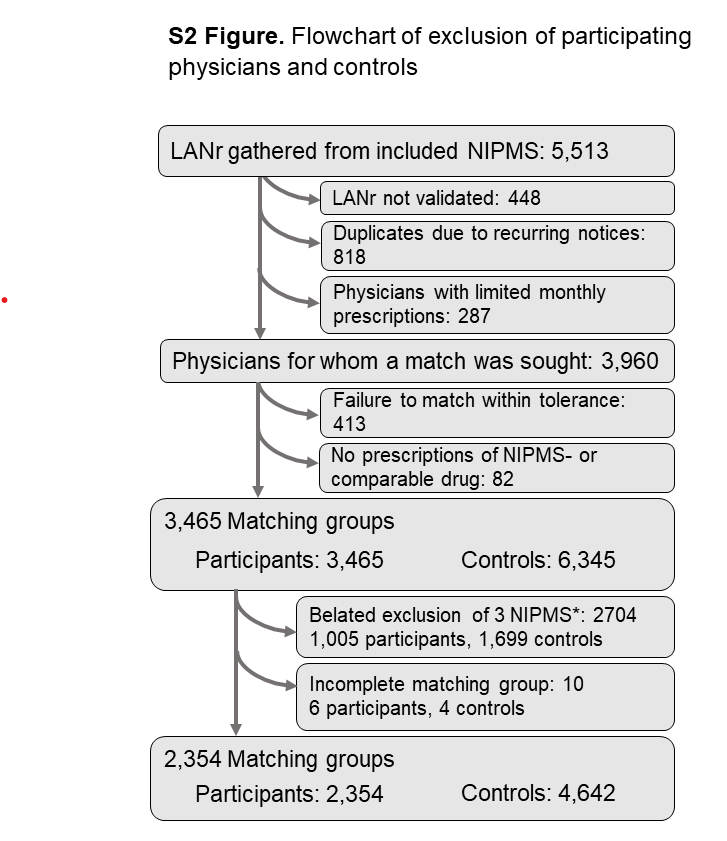

Supplement: S2 Fig — (TIF) [file pmed.1003151.s006.tif]
