# Supplementary material for: Impact of physicians’ participation in non-interventional post-marketing studies on their prescription habits: A retrospective 2-armed cohort study in Germany
Source: PLoS Med. 2020 Jun 26;17(6):e1003151. doi: 10.1371/journal.pmed.1003151 (PMC7319278; doi:10.1371/journal.pmed.1003151)
Supplement: S1 File — Request regarding the use of privacy-protected data for the purpose of this study. (DOCX) [file pmed.1003151.s007.docx]

| Prof. Dr. Ralf Kölbel Geschw.-Scholl-Platz 1· 80539 München | | **Prof. Dr . Ralf Kölbel**  Sekretariat: +49 (0)89 2180-2849  Fax: +49 (0)89 2180-3580  sekretariat.koelbel@jura.uni-muenchen.de  www.lmu.de |  |
| --- | --- | --- | --- |
| Bundesministerium für Gesundheit  Herrn Dr. Ulrich Orlowski  Abteilungsleiter 2 “Gesundheitsversorgung, Krankenversicherung“  Friedrichstr. 108  10117 Berlin | |  |  |
|  |  |  | München, 14.04.2020 |

**Antrag** auf Genehmigung der **Verwendung von Sozialdaten** für die Forschung gem. § 75 Abs. 2 SGB X; hier: Verwendung der beim GKV-Spitzenverband gem. § 67 Abs. 6 AMG, gem. § 63f Abs. 4 AMG und gem. § 84 Abs. 5 SGB V erfassten Daten für **Forschung zur Korruption im Gesundheitssystem**

Sehr geehrter Herr Dr. Orlowski,

hiermit stelle ich – in meinem eigenen Namen und im Namen von Prof. Dr. Klaus Lieb (Universität Mainz) – einen Antrag auf Verwendung von Sozialdaten für die wissenschaftliche Forschung im Sozialleistungsbereich gem. §§ 75 Abs. 1 Nr. 1, Abs. 2 SGB X und bitte um entsprechende Genehmigung. Sollte das Genehmigungsverfahren an das Bundesversicherungsamt übertragen worden sein, bitte ich um entsprechende Weiterleitung des Antrages.

**I. Allgemeine Begründung**

1. Bei den benötigten Sozialdaten handelt es sich um Verschreibungs- und ggf. auch um Einzelangaben einer bestimmten natürlichen Person (Betroffener), die vom GKV-Spitzenverband (im Folgenden: GKV-SV) erhoben, verarbeitet oder genutzt werden (§ 67 Abs. 1 Satz 1 SGB X). Konkret beantragt wird die Verwendung von Verschreibungsdaten nach § 84 Abs. 5 SGB V (GAmSi-Daten) sowie die Verwendung der beim GKV-SV gem. § 63f Abs. 4 und § 67 Abs. 6 AMG meldepflichtigen Arztnummern von Ärzten, die Leistungen zu Lasten der gesetzlichen Krankenversicherung erbringen und an einer bestimmten Gruppe von Anwendungsbeobachtungen/nichtinterventionellen Unbedenklichkeitsprüfungen teilnehmen. Der Antrag bezieht sich ferner auf die Ermittlung von Arztnummern anhand des Arztstammdatenverzeichnis (§ 293 Abs.4 SGB V ).

2. Die Verwendung dieser Sozialdaten ist für ein Vorhaben der wissenschaftlichen Forschung im Sozialleistungsbereich erforderlich, das durch Prof. Lieb und mich konzipiert worden ist und durch den GKV-SV organisatorisch unterstützt wird. Wegen der Einzelheiten dieses wissenschaftlichen Forschungsvorhabens – d.h. wegen dessen Beschreibung sowie der Vorhabensphasen und Zeiträume, für die die besagten Sozialdaten benötigt werden – verweise ich auf die (unter Punkt II - III) folgende Projektdarstellung.

Bei dem von uns durchgeführten Forschungsprojekt handelt es sich um wissenschaftliche Forschung, die Erkenntnisse erwarten lässt, die für den Sozialleistungsbereich, d.h. für die Verwirklichung der sozialen Rechte gem. § 2 Abs. 1 Satz 1 SGB I i.V.m. § 4 SGB I von Bedeutung sein können. Insbesondere ist mit Ergebnissen zu folgenden Fragen zu rechnen: Werden Anwendungsbeobachtungen/nichtinterventionelle Unbedenklichkeitsprüfungen durch Arzneimittelhersteller in korruptionsnaher Weise und / oder zur Förderung ihres Produktvertriebes eingesetzt? Haben Anwendungsbeobachtungen/nichtinterventionelle Unbedenklichkeitsprüfungen (potenziell kostenrelevante) Auswirkungen auf das Verschreibungsverhalten der teilnehmenden Ärzte? Unterscheiden sich die Auswirkungen von korruptionsnah gestalteten Anwendungsbeobachtungen von den Auswirkungen, die möglicherweise auch regulär (also nicht als Marketing-Tool) ausgestaltete Anwendungsbeobachtungen/nichtinterventionelle Unbedenklichkeitsprüfungen auf das Verschreibungsverhalten der hieran teilnehmenden Ärzte haben?

3. Die Erforderlichkeit der Verwendung der besagten Sozialdaten wird im Anschluss an die Darstellung des Projektvorhabens begründet (unten IV.).

**II. Projektdarstellung**

1. Vorbemerkungen:

Das Thema „Anwendungsbeobachtungen“ (im Folgenden AWB) ist für die gesetzliche Krankenversicherung von besonderer Aktualität und praktischer Bedeutung. Der Regierungsentwurf für das inzwischen in Kraft getretene Gesetz zur Bekämpfung von Korruption im Gesundheitswesen führt in der Gesetzesbegründung für einen neuen Straftatbestand der Bestechlichkeit und Bestechung im Gesundheitswesen u.a. aus: „Straflosigkeit besteht freilich nicht, wenn die Anwendungsbeobachtung Bestandteil einer Unrechtsvereinbarung ist und die vorgesehene Vergütung den teilnehmenden Arzt nicht für seinen zusätzlichen Aufwand entschädigt, sondern ihm tatsächlich als Bestechungsgeld für die bevorzugte Verordnung bestimmter Präparate und damit für eine unlautere Bevorzugung des Vorteilsgebers gewährt wird. Anhaltspunkte für eine strafbare Unrechtsvereinbarung können sich insbesondere daraus ergeben, dass der Entschädigung keine erkennbare ärztliche Gegenleistung gegenübersteht oder die Entschädigung den geleisteten Aufwand deutlich übersteigt. In der Vergangenheit haben sich gerade vertragliche Vereinbarungen über die Durchführung von Anwendungsbeobachtungen als Möglichkeit für eine korruptive Einflussnahme auf das Verschreibungsverhalten von Ärzten erwiesen.“ (BT-Drs. 18/6446, S. 19; dazu auch BT- Drs 18/8056, S. 8).

Das Vorhaben knüpft an diese Hinweise an. Es steht hierbei in einem Zusammenhang mit einem DFG-geförderten Forschungsprojekt „Compliance in Practice. Unternehmerische Anpassungsprozesse an Kriminalisierungsverläufe am Beispiel des sog. Pharmamarketings“, das ich mit Prof. Theile (Universität Konstanz) seit 2014 durchführe.^[[1]](#footnote-1)^ Dabei geht die hier beantragte Untersuchung (wie der Gesetzgeber) von der Annahme aus, dass ungeachtet der zwischenzeitlich erfolgten regulatorischen Maßnahmen weiterhin auch solche Anwendungsbeobachtungen/nichtinterventionelle Unbedenklichkeitsprüfungen durchgeführt werden, die fragwürdig sind – also AWB, bei denen es sich um Zweckentfremdungen handelt und bei denen der Marketing- bzw. Verschreibungsförderungseffekt im Vordergrund steht.^[[2]](#footnote-2)^ Eine Studie, die dieser Frage empirisch nachgeht, würde den öffentlichen und wissenschaftlichen Diskurs zu problematischen Ausprägungen des sog. Pharmamarketings fördern, zumal zu dieser Fragestellung bislang nur Analysen mit beschränkter Aussagekraft vorliegen.^[[3]](#footnote-3)^ Zu diesem Zweck soll anhand von AWB-Meldungen erstmals (!) der Nachweis geführt werden, dass solche oder jedenfalls entsprechend auffällige AWB auftreten und dass diese sich sodann im Verschreibungsverhalten der teilnehmenden Ärzte niederschlagen, und zwar anders (stärker) als unauffällige bzw. regulär durchgeführte AWB. Aber auch die gleichermaßen untersuchte Frage, ob nicht auch regelgerecht durchgeführte Anwendungsbeobachtungen/nichtinterventionelle Unbedenklichkeitsprüfungen das Verschreibungsverhalten der Teilnehmer durchaus beeinflussen, betrifft eine Problemstellung, die für gesundheitspolitische Entscheidungen klärungsbedürftig ist.

Der GKV-SV hat die Bereitschaft erklärt, das Projekt aktiv zu unterstützen und bei Vorliegen der hier beantragten datenschutzrechtlichen Genehmigung die Bereitstellung der projektnotwendigen Daten sicherzustellen.

2. Projektphase I: Identifizierung auffälliger AWBs, bei denen Marketingzwecke im Vordergrund stehen

Die Abgrenzung von AWB (bzw. nichtinterventionellen Studien nach Zulassung des Präparates) und klinischen Phase IV-Studien ist nicht immer klar. Das Gesetz sieht das wesentliche Unterscheidungsmerkmal darin, dass die beobachtete Behandlung bei den AWB allein die der „normalen“ ärztlichen Praxis ist und nicht (wie bei klinischen Studien) anhand eines Untersuchungsplanes erfolgt (§ 4 Abs. 13 S. 1 und 3 AMG). Allerdings sind klinische Studien registrierungs- und genehmigungspflichtig, während das Gesetz für AWB lediglich eine Anzeigepflicht vorsieht (§§ 63f Abs. 4 und 67 Abs. 6 AMG). Für das Projekt bedeutet dies, dass die Einordnung durch das pharmazeutische Unternehmen maßgebend ist: Weil die Anmeldung nach § 63f Abs. 4 bzw. § 67 Abs. 6 AMG erfolgt ist, wird unterstellt, dass es sich um eine AWB und nicht um eine klinische Studie in Phase IV handelt.

Dem GKV-SV werden durch Unternehmen, die eine AWB durchführen, gem. § 63f Abs. 4 bzw. § 67 Abs. 6 AMG folgende Angaben gemeldet: Ort, Zeit, Ziel und Beobachtungsplan bzw. Protokoll der Anwendungsbeobachtung/nichtinterventionellen Unbedenklichkeitsprüfung, die beteiligten Ärzte namentlich mit Angabe der lebenslangen Arztnummer, Art und Höhe der jeweils an sie tatsächlich geleisteten Entschädigungen, eine Ausfertigung der mit ihnen geschlossenen Verträge und jeweils eine Darstellung des Aufwandes für die beteiligten Ärzte und eine Begründung für die Angemessenheit der Entschädigung.^[[4]](#footnote-4)^ Da Hersteller und Präparat in der Regel bekannt sind, stehen überdies einige „Kontextinformationen“ zur Verfügung. Anhand dieser Informationen wird eine Stichprobe von 138 AWBs einer „Auffälligkeitsbewertung“ unterzogen. Dies geschieht einmal anhand von Kriterien, anhand derer die formale und inhaltliche Richtlinienkonformität^[[5]](#footnote-5)^ der AWBs eingestuft und die bereits vorliegenden Studien zu Qualität und Problemen deutscher AWB^[[6]](#footnote-6)^ repliziert werden können. Darüber hinaus werden weitere Merkmale der AWB erhoben, die als Indikatoren für einen etwaigen Marketingzweck fungieren (z.B. aufwandsüberschreitende Vergütungshöhe^[[7]](#footnote-7)^, geringer ärztlicher Beteiligungsaufwand, weit zurückliegender Zeitpunkt der Markteinführung des Arzneimittels, auffällige Marktentwicklung des Medikamentes, fehlender inhaltlicher Abschlussbericht bei der zuständigen Bundesoberbehörde). Anhand der jeweils vorliegenden Merkmale (die je nach ihrer marketingbezogenen Indizstärke in gewichteter Weise berücksichtigt werden) erfolgt schließlich eine Einstufung der untersuchten AWB in drei Gruppen („unauffällig“, „auffällig“ und „sehr auffällig“). Abgesehen von den frei recherchierbaren Kontextinformationen werden die erforderlichen Informationen durch den GKV-SV aus dessen AWB-Meldungsdatenbank zur Verfügung gestellt. Dies geschieht auf Grundlage des Informationsfreiheitsgesetzes.

Hinweis: Da hier keine personenbezogenen Daten eingeschlossen sind – bspw. werden die Anzahl und die Vergütung der teilnehmenden Ärzte ohne jede Zuordnung zu den anonym bleibenden Teilnehmer/innen und allein anhand der jeweiligen Liste erhoben –, bedarf es nach unseren Bewertung keiner speziellen datenschutzrechtlichen Genehmigung. Dies gilt insbesondere deshalb, weil die personenbezogenen Informationen über die ärztlichen AWB-Teilnehmer (Arztnummer, Name usw.) durch den GKV-SV gesperrt bleiben. Bedarf an diesen Informationen besteht (die Genehmigungserteilung vorausgesetzt) erst in der zweiten Projektphase – und sie werden auch erst für diese verwendet.

3. Projektphase II: Ermittlung und Auswertung des Verschreibungsverhaltens von AWB-Teilnehmern

a) Für die Teilnehmer der Anwendungsbeobachtungen soll das Verschreibungsverhalten bzgl. des Medikamentes, das Gegenstand der AWB war, sowie der Medikamente aus der gleichen Substanzklasse (bis zum 4. Level des ATC-Codes), ausgewertet werden. Insbesondere geht es hierbei darum, etwaige Veränderungen zu dokumentieren. Konkret untersucht werden soll die Häufigkeit, mit der die betreffenden Ärzte das Medikament aus der AWB im Zeitraum t1 (= im Jahr vor der AWB-Teilnahme), im Zeitraum t2 (= während der AWB-Teilnahme) und im Zeitraum t3 (= im Jahr nach der AWB-Teilnahme) verschrieben haben. Zusätzlich untersucht werden soll eine eventuell daraus resultierende Verschiebung des Verschreibungsverhaltens innerhalb der Substanzklasse des AWB-Medikamentes. Erfasst werden die betreffenden Verschreibungsdaten im Rahmen des GAmSi-Projektes gem. § 84 Abs. 5 SGB V ebenfalls durch den GKV-SV. Konkret vorgehalten werden dort derzeit die Daten von 2012 bis zum aktuellen Zeitpunkt.

Es ist indes nicht möglich, das Verschreibungsverhalten für die Teilnehmer aller AWB auszuwerten. Bei dieser Auswertungsphase nicht berücksichtigt werden

- AWB, die vor dem 1.1.2013 begannen (weil bei diesen AWB die t1-Phase ganz oder teilweise vor 2012 liegt und hierfür – wie eben erwähnt – im GAmSi-System keine Verschreibungsdaten vorhanden sind),
- AWB, die nicht-verschreibungspflichtige Medikamente zum Gegenstand haben (weil hier keine Verschreibungsdaten anfallen) und
- AWB, an denen allein Krankenhausärzte teilnehmen (weil hier keine registrierten Verschreibungen zu erwarten sind).^[[8]](#footnote-8)^

Von den in Phase I ausgewerteten AWB erfüllen 28 Studien diese Bedingungen. Um die Aussagekraft der Ergebnisse zu erhöhen, haben wir vorgesehen, bei allen weiteren (nicht in Phase I berücksichtigten) AWB, die den genannten Kriterien entsprechen, eine Nacherhebung vorzunehmen und in die Auswertungen von Phase II eingehen zu lassen. Im Ergebnis bezieht sich Phase II daher auf 51 AWB mit insg. ca. 4.900 Teilnehmern. Darunter befinden sich unauffällige, auffällige und sehr auffällige AWB.

b) Vorgesehen ist, die Verschreibungszahlen in t1 mit denen in t2 und t3 zu vergleichen. Erfolgen soll dies auf der Ebene der Gesamtgruppe (= Teilnehmer aller einbezogenen AWB zusammen), auf der Ebene der Fachgruppe (=Teilnehmer aller einbezogenen AWB, die nach medizinischen Bereichen zusammengefasst und unterschieden werden) und auf der Ebene der Auffälligkeitsgruppe (= Teilnehmer aller einbezogenen AWB, die nach ihrem Auffälligkeitsgrad gem. Phase I zusammengefasst und unterschieden werden). Dabei erwarten wir, dass auf allen drei Ebenen die Verschreibungszahlen in t1 am niedrigsten und in t2 am höchsten sind. Ferner erwarten wir, dass die Differenzen zwischen t1 und t2 sowie auch t3 bei der Teilgruppe der Teilnehmer an sehr auffälligen AWB stärker ausgeprägt sind als in den beiden anderen Teilgruppen und auch in der Gesamtgruppe. Ferner erwarten wir fachgruppenbezogene Unterschiede in den besagten Differenzen (wobei wir an diesem Punkt induktiv vorgehen und keine Annahmen formulieren, bei welchen medizinischen Bereichen die Unterschiede am deutlichsten sein werden).^[[9]](#footnote-9)^

c) Soweit die erwarteten Differenzen bestätigt werden sollten, könnte dies allerdings durchaus auf externen Gründen beruhen (bspw. auf Veränderungen im jeweiligen Patientenstamm oder auf Marktveränderungen). Dennoch wäre zumindest bei klaren Unterschieden eine andere Interpretation (nämlich die Rückführung auf einen Marketingeffekt) plausibler (v.a. wenn die höchsten Verschreibungszahlen tatsächlich in t2 fielen). Um diese Schlussfolgerung indes noch weiter abzusichern und um AWB-bedingte und allgemeine (AWB-unabhängige) Verordnungstrends methodisch klar zu unterscheiden, sollen zwei Vergleichsverfahren durchgeführt werden:

- Ein erster Vergleich ist vorgesehen mit der Entwicklung der Gesamtverordnungsmengen der AWB-gegenständlichen Medikamente. Diese müssen für die jeweiligen Phasen t1, t2 und t3 aller AWB ermittelt werden.^[[10]](#footnote-10)^ Dies erlaubt sodann abermals einen Vergleich auf allen drei (unter dem Punkt b) genannten) Ebenen. Dabei erwarten wir, dass der Verschreibungszuwachs in t2 (und ggf. auch in t3) bei der Gesamtgruppe aller AWB stärker ausfällt als in der Gesamtverschreibungsentwicklung, dies allerdings bei der Gruppe sehr auffälliger AWB (und bei AWB bestimmter medizinischer Fachbereiche) noch stärker.
- Ein zweiter Vergleich ist vorgesehen durch Bildung konkreter Vergleichsgruppen. Hiernach soll für jeden individuellen Teilnehmer einer (in Phase II eingeschlossenen) AWB ein „Zwilling“ in den GAmSi-Daten identifiziert werden (maßgeblich für die Paarbildung: gleiche Fachgruppenzugehörigkeit und gleiche Verschreibungshäufigkeit des jeweiligen Medikamentes in t1). Hierdurch entsteht für die Teilnehmergruppe jeder AWB eine Nichtteilnehmer-Vergleichsgruppe. Dies erlaubt sodann erneut auf allen drei (unter dem Punkt b) genannten) Ebenen einen Vergleich der Verschreibungsentwicklung. Dabei erwarten wir, dass der Verschreibungszuwachs in t2 (und ggf. auch in t3) bei der Gesamtgruppe aller AWB stärker ausfällt als in der Gesamtgruppe aller Vergleichsgruppen. Noch deutlichere Unterschiede sollten sich bei der Gegenüberstellung sehr auffälliger AWB mit deren jeweiligen Vergleichsgruppen sowie bei der Gegenüberstellung der AWB bestimmter medizinischer Fachbereiche mit deren jeweiliger Vergleichsgruppe ergeben.

d) Falls die Differenzen sich bestätigen, soll ferner anhand der Verschreibungsdaten der anderen Präparate innerhalb der Substanzklasse untersucht werden, ob die erwarteten Differenzen eine Verschiebung des Verschreibungsverhaltens hin zu den Präparaten, die in der AWB untersucht werden, darstellen, oder ob die Differenzen auf eine tatsächliche Mehrverschreibung hindeuten. Geklärt werden soll also, ob die AWB-Präparate anstelle ähnlich geeigneter Präparate oder zusätzlich zu den regulären Verschreibungen verschrieben werden (also in Fällen zum Einsatz kommen, in denen ohne AWB ggf. kein Medikament verschrieben worden wäre). Damit sollen etwaige Kosteneffekte analysiert werden, die für die Kostenträger durch die AWB-bedingten Änderungen im Verschreibungsverhalten eintreten (können).

**III. Technische Realisierung**

Die Umsetzung der Projektphase ist in folgenden Schritten vorgesehen:

1. Beim GKV-SV wird für uns ein Zugang zu den AWB-Meldungen gem. § 63f Abs. 4 und § 67 Abs. 6 AMG, zu den Arzneimittelverordnungsdaten nach § 84 Abs. 5 SGB V (GAmSi-Daten) für die spezifischen Zeiträume t1 bis t3 sowie zu dem Arztstammdatenverzeichnis nach § 293 Abs. 4 SGB V eingerichtet.
2. Sodann werden von uns für die in Phase II einbezogenen AWB anhand der Meldungen und unter Verwendung der lebenslangen Arztnummern Teilnehmerlisten erstellt. Für die teilnehmenden Ärzte, deren lebenslange Arztnummer nicht in den AWB-Meldungen enthalten ist, wird die Arztnummer durch uns mit Hilfe des Arztstammdatenverzeichnisses festgestellt.
3. Anhand der ausgewählten AWB-Teilnehmerlisten werden in den GAmSi-Daten die Verschreibungszahlen für jeden Teilnehmer, und zwar monatsgenau für die AWB-spezifischen Gesamtphasen t1 bis t3. ermittelt
4. Sodann werden auf Grundlage der Verordnungsprofile der AWB-Teilnehmer in den GAmSi-Daten die oben beschriebenen Zwillinge/Matching-Partner (vgl. II.3.c) mit den Kriterien identifiziert und analog deren Verschreibungszahlen monatsgenau für die jeweiligen Zeiträume t1 bis t3 abgefragt. Diese werden anschließend zu AWB-konkreten Vergleichsgruppen zusammengefasst.
5. Aus den Verschreibungszahlen der AWB-Teilnehmer und deren Zwillingen/Matching-Partnern werden die strukturierten Auswertungsdaten erzeugt. Der Auswertungsdatensatz wird zudem einer Anonymisierung unterzogen, da eine weitere Verknüpfung über die lebenslange Arztnummer in dieser Phase nicht mehr erforderlich ist.
6. An den so ermittelten Auswertungsdaten nehmen wir deren statistische Auswertung in der oben skizzierten Form vor.

In Punkt 2. der obigen Schrittfolge ist eine Komplikation berücksichtigt, die sich bei der Umsetzung der Analyseschritte voraussichtlich ergeben wird. Dies betrifft namentlich den folgenden Punkt: Nach Auskunft des GKV-SV erfüllen nicht wenige Unternehmen ihre Meldepflicht gem. § 63f Abs. 4 bzw. § 67 Abs. 6 AMG nur mangelhaft und geben für die teilnehmenden Ärzte lediglich Name und Anschrift, aber keine oder fehlerhafte Arztnummern an. Bei diesem Verhalten ist es offenbar teilweise auch geblieben, obwohl der GKV-SV zwischenzeitlich die fehlenden Angaben nachgefordert hat bzw. derzeit nachfordert. Im Rahmen unseres Projektes bedarf es in diesen Fällen daher eines zusätzlichen Arbeitsschrittes, damit die AWB-konkreten Teilnehmerlisten durch den GKV-SV gebildet werden können. Die lebenslange Arztnummer muss hier anhand der gemeldeten Namens- und Adressdaten erst ermittelt werden. Das erfolgt mit Hilfe des Arztstammdatenverzeichnisses (vgl. § 293 Abs. 4 SGB V). Dieses Vorgehen ist aus unserer Sicht nicht verzichtbar, da die Aussagekraft der Ergebnisse sonst beeinträchtigt wäre. Ohne die Ermittlung der Arztnummern könnte nämlich bei schätzungsweise 1.300 Teilnehmern/innen das Verschreibungsverhalten nicht ausgewertet werden.^[[11]](#footnote-11)^ Auch müssten wir davon ausgehen, dass sich ca. 10 AWB nicht in Phase II einbeziehen ließen, weil bei ihnen mehr als 20 % der Teilnehmer ausfallen. Dass es sich bei nicht auswertungsfähigen Teilnehmern/innen bzw. AWB gerade um solche handelt, bei denen auffällige Ergebnisse zu erwarten wären, ist dabei durchaus zu vermuten. Insofern handelt es sich bei der Arztnummern-Feststellung um einen zentralen Schritt.

**IV. Erforderlichkeit der dargestellten und beantragten Datenverwendung**

1. Fehlen von Alternativen

Die Erforderlichkeit der geschilderten Verwendung der Sozialdaten ergibt sich daraus, dass das Forschungsvorhaben auf andere Weise nicht erfüllt werden kann und die Nutzung ein geeignetes Mittel ist, für das keine zumutbaren Alternativen bestehen. Alternativ nutzbare Daten sind nicht bereits bei anderen Stellen in ausreichendem Umfang und ausreichender Aktualität vorhanden. Das Forschungsziel kann auch nicht mit alternativen Erhebungsmethoden erreicht werden.

Feststellungen über das Verschreibungsverhalten von Ärzten (und über dessen Veränderung während oder nach der Teilnahme an einer Anwendungsbeobachtung) sind ausschließlich anhand der Verschreibungsdaten möglich, die beim GKV im Rahmen von § 84 Abs. 5 SGB V gespeichert werden. Um die Verschreibungsdaten jener Ärzte zu identifizieren, für die wegen ihrer Teilnahme an Anwendungsbeobachtungen/nichtinterventionellen Unbedenklichkeitsprüfungen die besagten Informationen benötigt werden, ist es wiederum erforderlich, dass Arztnummernstammverzeichnis für eine möglichst vollständige Zuordnung und Überprüfung der gemeldeten lebenslangen Arztnummern zu nutzen. Eine weitergehende Verwendung ist nicht erforderlich und wird auch nicht erfolgen.

Insbesondere liegt eine Alternative zur hier beantragten Datenverwendung nicht etwa darin, die besagten Ärzte vorab um ihre Genehmigung zu bitten. Abgesehen davon, dass für das Einholen der Genehmigungen ein eigenständiger Gebrauch personalisierter Daten erforderlich wäre (d.h. die Nutzung der notwendigen Kontaktdaten), muss befürchtet werden, dass ein erheblicher Anteil der Anfragen negativ beschieden würde. Es ist insbesondere zu vermuten, dass dies zu einer Verzerrung der Stichprobe (Selektionsbias) führen würde, da vermutlich eher Ärzte zustimmen würden, die geringere Honorare erhalten haben. Für diese Annahme spricht die eingeschränkte Bereitschaft vieler Ärzte, ihre von der pharmazeutischen Industrie erhaltenen Zuwendungen im Zuge der sog. Transparenzinitiative offenzulegen (vgl. Osterloh, Dtsch Arztebl 2016; 113(39): A-1690: nur ca. ein Drittel hat zugestimmt). Es ist mit anderen Worten davon auszugehen, dass die Abstimmung mit den fraglichen Ärzten ein erhebliches Risiko dafür birgt, dass es zu einer Verzerrung der Forschungsergebnisse kommt (zu diesem Aspekt vgl. nur Rombach, in: Hauck/Noftz, SGB X, § 75, Rz. 38a, 40b). Die Ziele unseres wissenschaftlichen Vorhabens wären dadurch nicht mehr erreichbar.

2. Überwiegendes Interesse

Der Übermittlung der Sozialdaten stehen keine schutzwürdigen Interessen der Betroffenen entgegen. Ein Bezug zur Intimsphäre ist ersichtlich nicht gegeben. Selbst wenn schutzwürdige Interessen der Betroffenen tatsächlich beeinträchtigt wären, stünden diese der hier beantragten Datenübermittlung nicht entgegen, da das öffentliche Interesse an dem beantragten Forschungsvorhaben das allenfalls marginal berührte Geheimhaltungsinteresse der betroffenen Ärzte erheblich überwiegt.

Das Vorhaben zielt auf die Untersuchung von Mechanismen korruptionsnaher Kooperationsformen im Gesundheitssystem. In dieser Hinsicht hat der Gesetzgeber durch Einführung der §§ 299a ff. StGB dokumentiert, dass Missständen mit Blick auf ganz erhebliche gesellschaftliche Belange (namentlich den Wettbewerbsschutz und das Vertrauen in die medizinischen Leistungserbringer) entschieden entgegenzutreten ist. Zugleich aber ist der Mangel an gesichertem empirischem Wissen hinsichtlich der Problemprävalenz, aber auch mit Blick auf die fraglichen Mechanismen und Wirkweisen evident. Hier im Teilbereich der Anwendungsbeobachtungen/nichtinterventionellen Unbedenklichkeitsprüfungen für die erforderlichen, bislang (in der Bundesrepublik) aber nahezu vollständig fehlenden Erkenntnisse zu sorgen, ist das Ziel des Projekts.

In diesem Zusammenhang ist noch einmal auf die Einführung und Erweiterung der § 63f Abs. 4 und § 67 Abs. 6 AMG hinzuweisen – jene Regelung, mit der pharmazeutische Unternehmen, die Anwendungsbeobachtungen/nichtinterventionellen Unbedenklichkeitsprüfungen durchführen, dazu verpflichtet wurden, u.a. die Namen der teilnehmenden Ärzte und die ihnen gezahlte Vergütung an den GKV (und andere Institutionen) mitzuteilen. Damit hat man ausdrücklich (!) die Absicht verfolgt, missbräuchliche Gestaltungen zu verhindern und durch meldepflichtige Informationen im Bereich von Anwendungsbeobachtungen für Transparenz zu sorgen (dazu sehr klar BT-Drs. 17/13770, S. 20; vgl. auch BT- Drs. 18/8056, S. 2). Insofern hat der Gesetzgeber selbst schon eine generalisierende Interessenabwägung bzgl. einer Verwendung der gemäß § 63f Abs. 4 und § 67 Abs. 6 AMG gemeldeten Daten vorgenommen: Sofern deren Verwendung zu wissenschaftlichen Zwecken geschieht, haben die damit verfolgten Belange wesentlich größeres Gewicht als die Interessen, die die betroffenen Personen an einer Nicht-Verwendung der gemeldeten Angaben haben kann.

3. Abschließende Erwägungen

Soweit die AWB-Meldungen und die Verschreibungsdaten durch Nutzung der gemeldeten (und teilweise festgestellten) Arztnummern miteinander verbunden werden, handelt es sich um einen Analysevorgang, dessen Vornahme durch den Gesetzgeber eigens ermöglicht worden ist, nämlich durch Einführung der entsprechenden AWB-Meldepflichten. Datenschutzrechtlich weist diese Analyse aus meiner Sicht daher keine weitergehende Belastungswirkung auf. Dem Umstand, dass die Feststellung der Verschreibungsdaten der AWB-Teilnehmer nicht durch den Meldungsadressaten (den GKV-SV), sondern durch uns erfolgt, wird dadurch Rechnung getragen, dass wir die Verschreibungsdaten anhand der Arztnummern (also pseudonomysiert) erheben und dass die ggf. zur Arztnummernfeststellung erforderlichen Individualdaten vor (!) der Verschreibungsdatenerhebung an den GKV-SV zurückgegeben werden. Im Übrigen handelt es sich – das sei nachdrücklich betont – in keiner Phase der Erhebung um Daten mit Patientenbezug.

Für den Fall einer abweichenden rechtlichen Bewertung weise ich ergänzend darauf hin, dass die Ermittlung der Verschreibungsdaten nicht notwendigerweise durch uns erfolgen muss, sondern auch durch den GKV-SV durchgeführt und sodann an uns übermittelt werden könnte. Angesichts dessen beantrage ich hilfsweise (!), das folgende Vorgehen zu genehmigen: Das Team von Prof. Lieb und mir erstellt die Arztnummern-Listen der AWB-Teilnehmer und nimmt anhand des Arztstammdatenverzeichnisses die dafür erforderlichen Ergänzungen vor. Abweichend von der in III. dargestellten Schrittfolge werden die Verschreibungsdaten der Teilnehmer anhand dieser Listen allerdings durch den GKV-SV in der geschilderten Weise bestimmt und uns pseudonomysiert (d.h. arztnummern-bezogen) zur Verfügung gestellt. Dies gilt auch für die Ermittlung der Zwillinge/Matching-Partner und ihrer Verschreibungsdaten.

Was die teilweise erforderliche Nutzung des Arztstammdatenverzeichnisses zur Feststellung der Arztnummern betrifft (oben III. am Ende), muss auf folgende Gesichtspunkte hingewiesen werden:

- Dieser Schritt ist nur deshalb erforderlich, weil manche meldepflichtigen Unternehmen ihre Verpflichtung unzulänglich erfüllen und dem GKV-SV die mitteilungsbedürftigen Arztnummern gleichsam vorenthalten oder fehlerhaft gemeldet haben (zur Häufigkeit oben III. am Ende).
- Würde uns die Möglichkeit verwehrt, die Arztnummern in der besagten Weise festzustellen, wäre das Verschreibungsverhalten der betr. AWB-Teilnehmer in vielen Fällen nicht analysierbar. Gesetzgeberisch gewollte (!) Untersuchungen der vorliegenden Art könnten durch die meldepflichtigen Unternehmen also im Wege unvollständiger Meldungen verhindert werden. Eine Befugnis des GKV-SV, die Unternehmen zur Nachbesserung der Meldung nicht nur aufzufordern, sondern auch juristisch zu zwingen, existiert im Rahmen von § 63f Abs. 4 und § 67 Abs. 6 AMG nämlich nicht.

Die einfachere und aus Sicht des Datenschutzes womöglich vorzugswürdige Option, bei der der GKV-SV die fehlenden Arztnummern anhand des Arztstammdatenverzeichnisses selbst ermittelt und auf diese Weise die defizitären AWB-Meldungen ergänzt, bevor die hier beantragte Untersuchung beginnt, ist durch § 293 Abs. 4 SGB V verschlossen.^[[12]](#footnote-12)^ Deshalb wird hier beantragt, dass die Feststellung der Arztnummern anhand des Arztstammdatenverzeichnisses durch uns selbst durchgeführt werden darf. Die Rechtsgrundlage hierfür ist § 75 Abs. 2 SGB X (speziell zur Zweckänderung vgl. § 67c Abs. 2 Ziff. 3 SGB X). Um eine Nutzung der Namens- und Adressdaten, die über die Arztnummernfeststellung hinausgeht, zu verhindern, würden wir diesen Arbeitsschritt in den Räumlichkeiten des GKV-SV vornehmen und eine sofortige Datenrückgabe sicherstellen. Zu Begründung verweisen wir darauf, dass andernfalls eine Untersuchung, die augenscheinlich den gesetzgeberischen Interessen und Anliegen entspricht, in ihrer Aussage gefährdet wäre. Im Übrigen wurde durch die Arzneimittelkommission der deutschen Ärzteschaft, namentlich durch deren Vorsitzenden Wolf-Dieter Ludwig, erst kürzlich wieder betont, dass eine unabhängige wissenschaftliche Untersuchung und Bewertung von Anwendungsbeobachtungen erforderlich ist.

Prof. Dr. Ralf Kölbel

1. Für Zwischenergebnisse vgl. Kölbel, Zeitschrift für Internationale Strafrechtsdogmatik 2016, 452 – 466; Kölbel, in: Neubacher, F. (Hrsg.): Krise, Kriminalität, Kriminologie, Mönchengladbach 2016, S. 317 – 326; Kölbel/Herold/Lubner, in: Kubiciel/Hoven(Hrsg.): Korruption im Gesundheitswesen, 2016, S. 193 – 222. [↑](#footnote-ref-1)
2. Vgl. dazu bspw. den Erfahrungsbericht, den ein Unternehmensinsider zu derartigen Zweckentfremdungen vorgelegt hat: British Medical Journal (2012), ‘Post-Marketing Observational Studies: My Experience in the Drug Industry’, (http://dx.doi.org/10.1136/bmj.e3990.). Vgl. auch Gregor-Patera/Schader/Wild, Nicht-Interventionelle Studien (NIS) in Österreich. Systematische Analyse. Rapid Assessment Nr. 7c, 2016 sowie für Deutschland Grill, Süddeutsche Zeitung v. 10.03.2016, S. 16 und https://correctiv.org/recherchen/euros-fuer-aerzte/datenbank/ [↑](#footnote-ref-2)
3. Die Begrenztheit gilt auch für die jüngst vorgestellte Studie von Transparency International (vgl. etwa http://www.gesundheitsstadt-berlin.de/transparency-kritisiert-anwendungsbeobachtungen-6417/ sowie Spelsberg u.a., BMJ 2017;356:j337 http://dx.doi.org/10.1136/bmj.j337). [↑](#footnote-ref-3)
4. Meldemaske unter: http://www.gkv-spitzenverband.de/krankenversicherung/arzneimittel/ anzeige_einer_anwendungsbeobachtung/anzeige_einer_anwendungsbeobachtung.jsp. [↑](#footnote-ref-4)
5. Die Studie übernimmt die bei v. Jeinsen/Sudhop, Eur J Clin Pharmacol (2013) 69:1453–1466 genutzten Kriterien. Vgl. dazu ferner die Empfehlungen des Bundesinstituts für Arzneimittel und Medizinprodukte und des Paul-Ehrlich-Instituts zur Planung, Durchführung und Auswertung von Anwendungsbeobachtungen vom 7. Juli 2010; ferner: Sickmüller/Breitkopf, Pharm. Ind. 71, Nr. 5, 764–769 (2009) sowie http://www.fsa-pharma.de/verhaltenskodizes/fachkreise/#c84 (dort in § 19). Hierzu auch Eberhard et al., Pharm. Ind. 68, Nr. 5, 542−550 (2006); Theobald et al., GMS German Medical Science 2009, Vol. 7; Hochhauser, J. Clinical Research Best Practices, Vol. 5, No. 6, June 2009; Wink, Anwendungsbeobachtung in der ärztlichen Praxis, 2. Aufl. 2010; Klümper, pharmazeutische medizin 2011, 12-16. [↑](#footnote-ref-5)
6. Vgl. neben v. Jeinsen/Sudhop, Eur J Clin Pharmacol (2013) 69:1453–1466 auch Dietrich, PharmacoEconomics – German Research Articles 2009; 7 (1), 3-14; Hasford/Lamprecht, Eur J Clin Pharmacol (1998) 53: 369-371. [↑](#footnote-ref-6)
7. Dazu empirische Befragungsdaten bei Ruppert et al., GMS German Medical Science 2012, Vol. 10. [↑](#footnote-ref-7)
8. Eine weitere Einschränkung besteht darin, dass die AWB bis zu einem Zeitpunkt abgeschlossen wurden, der die Feststellung einer Verschreibungsentwicklung in t3 erlaubt. Dem wird dadurch Rechnung getragen, dass Studien, die über den 30.6.2015 hinaus laufen, bereits in Phase I nicht berücksichtigt wurden. [↑](#footnote-ref-8)
9. Ergänzend kann innerhalb einzelner AWB auch auf individueller Ebene geprüft werden, ob und wie stark sich die Verschreibungsentwicklung t1 bis t3 zwischen den Teilnehmern einer Studie unterschiedet. Dem läge die Annahme zugrunde, dass die Teilnehmer persönlich unterschiedlich stark für die Stimulierungswirkungen einer AWB-Teilnahme ansprechbar sind. [↑](#footnote-ref-9)
10. Die betreffenden Daten sind im Arzneiverordnungsreport für den gesamten relevanten Zeitraum für sämtliche verschreibungspflichtigen Präparate öffentlich zugänglich. [↑](#footnote-ref-10)
11. In einzelnen Fällen fehlen allerdings sämtliche Angaben zu den Teilnehmern, so dass eine Ermittlung der Arztnummern nicht möglich ist. Dies betrifft jedoch eine so geringe Zahl, dass die Aussagekraft der Ergebnisse hierdurch nicht gefährdet wird. [↑](#footnote-ref-11)
12. Dazu, dass der GKV-SV hierdurch darauf beschränkt wird, das ihm zugeleitete Arztstammdatenverzeichnis gleichsam als „blinde“ Datenweitergabe-Stelle an die Krankenkassen „durchzuleiten“, vgl. Hauck/Noftz SGB V § 293 Rn. 20. [↑](#footnote-ref-12)
