# Supplementary material for: Impact of physicians’ participation in non-interventional post-marketing studies on their prescription habits: A retrospective 2-armed cohort study in Germany
Source: PLoS Med. 2020 Jun 26;17(6):e1003151. doi: 10.1371/journal.pmed.1003151 (PMC7319278; doi:10.1371/journal.pmed.1003151)
Supplement: S2 File — (DOC) [file pmed.1003151.s008.doc]

STROBE Checklist for “Effect of physicians’ participation in non-interventional post-marketing studies on their prescription habits: A retrospective two-armed cohort study”

|  | Item No | Recommendation | Reported in section | Excerpt of Text |
| --- | --- | --- | --- | --- |
| **Title and abstract** | 1 | (*a*) Indicate the study’s design with a commonly used term in the title or the abstract | Title page | A retrospective two-armed cohort study |
|  |  | (*b*) Provide in the abstract an informative and balanced summary of what was done and what was found | Abstract, paragraph 2 | In a retrospective two-armed cohort study, the prescription behavior of 6,996 German physicians, of which 2,354 had participated in at least one of 24 NIPMS and 4,642 were controls, were analyzed. Data was acquired between Oct. 6th 2016 and June 8th 2018. Controls were matched by overall prescription volume and number of prescriptions of the drug studied in the NIPMS in the year prior to the NIPMS. Primary outcome was the relative rate of prescriptions of the drug studied in the NIPMS by participating physicians compared to controls during the NIPMS and the following year. Secondary outcomes were the proportion of prescriptions of the studied drug compared to alternative drugs used for the same indication, the revenue generated by these prescriptions, and the association between the marketing characteristics of the NIPMS and prescription habits. Physicians participating in an NIPMSprescribed more of the studied drug during and in the year after the NIPMS at a relative rate of 1.08 (95%-confidence interval: 1.07-1.10; p<0.001) and 1.07 (95%-CI: 1.05-1.09); p<0.001), respectively. Participating physicians were more likely than controls to prescribe one of the studied drugs rather than alternative drugs used for the same indication, OR: 1.039 (95% CI: 1.033-1.045). None of the marketing characteristics studied were significantly associated with prescription practices. |
| Introduction | | |  |  |
| Background/rationale | 2 | Explain the scientific background and rationale for the investigation being reported | Introduction,  Paragraphs 1 and 3 | Non-interventional post marketing studies (NIPMS) funded by the pharmaceutical industry have been subject of controversial debate.[…] Although several authors have argued that NIPMS mainly serve marketing purposes of the pharmaceutical industry, it has not yet been demonstrated that prescription behavior of physicians indeed changes during or after participation in NIPMS; such an effect has only been demonstrated for interventional studies (13–15). However, if this were the case, it would be an important reason to increase regulation of such studies. |
| Objectives | 3 | State specific objectives, including any prespecified hypotheses | Introduction, Paragraph 4 | The objective of the current study was thus primarily to investigate whether the participation in NIPMS affects the participating physicians’ prescription practices. In addition, we wanted to analyze whether such a possible change results in more expensive prescriptions by leading to a shift in prescriptions toward more expensive drugs used for the same indication. Lastly, we wanted to investigate whether certain characteristics of NIPMS are useful to predict the effect on the participating physicians’ prescriptions behavior. |
| Methods | | |  |  |
| Study design | 4 | Present key elements of study design early in the paper | Methods, subsection “Study Design”, Paragraph 1 | In a retrospective two-armed cohort study, we compared the prescription practices of physicians that had participated in a NIPMS with those of matched controls that had not participated in a NIPMS. After identifying eligible NIPMS, participating physicians were identified and matched 1:2 to control physicians, resulting in a “matching group”. |
| Setting | 5 | Describe the setting, locations, and relevant dates, including periods of recruitment, exposure, follow-up, and data collection | Methods, subsection “Data Sources and Setting”, Paragraphs 1 and 2 | “Data regarding the NIPMS as well as participating physicians were identified from notifications submitted to the National Association of Statutory Health Insurance Funds regarding NIPMS.[…] Data regarding the characteristics of the studies as well as the identifying data on physicians were gathered between Oct 6th 2016 and Jan 9th 2018. Because notifications were often incomplete or faulty regarding the identifying information on physicians, we conducted a validation by comparing the acquired data with a directory at the Innungskrankenkasse containing all practicing physicians in Germany. This validation process took place between Jan 22nd and 26th 2018. […] Prescription data were acquired using data from the GKV-Arzneimittelschnellinformation (GAmSI)-project, which consists of data reported to the National Association of Statutory Health Insurance Funds by pharmacies regarding filled prescriptions. […] Matching and acquiring the prescription data took place between January 27th and June 8th 2018.” |
| Participants | 6 | (*a*) Give the eligibility criteria, and the sources and methods of selection of participants. Describe methods of follow-up | Methods, subsection “NIPMS” paragraphs 1,2 | Eligible NIPMS were those that allowed us to study the prescription practices of the physicians between one year before and one year after the NIPMS. Criteria that enabled these analyses and reasons are given in table 1.[…] All physicians participating in one of the eligible NIPMS that could be identified using the data at the National Association of Statutory Health Insurance Funds and our validation process (see below) and had prescribed a minimum amount overall (to ensure they were still practicing) as well as of the drug studied in the NIPMS were included in the study. |
|  |  | (*b*) For matched studies, give matching criteria and number of exposed and unexposed | Methods, subsection “NIPMS” and “Bias”, S2 Appendix and S2 figure | “We matched two controls to each of these physicians using the number of overall prescriptions in packages as well as the number of defined daily doses (DDD) of the studied drug and alternative drugs in the year before the begin of the NIPMS (t0). Due to data privacy laws in Germany, other factors such as age, gender, location or specialization could not be considered for matching. Physicians participating in several different NIPMS were matched to different controls for each NIPMS (see S2 Appendix for exact process).” |
| Variables | 7 | Clearly define all outcomes, exposures, predictors, potential confounders, and effect modifiers. Give diagnostic criteria, if applicable | Methods, subsection “Outcome Measures”, paragraphs 1 and 2 | “Primary outcome was the relative rate of prescriptions of the drug studied in the NIPMS by participating physicians compared to their respective controls during the NIPMS and the year after.  Secondary outcomes were the proportion of prescriptions of the drug under study compared to alternative drugs, as well as the revenue generated by these prescriptions. In addition, the association between the marketing characteristics of the NIPMS and prescription habits was a secondary outcome.” |
| Data sources/ measurement | 8* | For each variable of interest, give sources of data and details of methods of assessment (measurement). Describe comparability of assessment methods if there is more than one group | Methods, subsection “Data Sources and Setting” | See above, “Setting” |
| Bias | 9 | Describe any efforts to address potential sources of bias | Methods, subsection “Bias”, paragraphs 1 and 2 | We used Directed Acyclic Graph modeling to identify the minimal sufficient adjustment set of covariates in the regression model to achieve unbiased estimation of the putative causal effect of participation in a NIPMS on prescription volume at t1 (18). If confounders have a direct effect on the exposure, but only an indirect effect on the outcome via prescription volume at t0, then matching on prescription volume at t0 is sufficient for unbiased estimation (see S1 figure for the directed acyclical graph model). Other confounders could only introduce bias through a direct effect on prescription volume at t1. Such a confounder would have to act differently on prescription volume at t1 compared to its effect on prescription volume at t0. This seems to be implausible for considered candidate confounders such as age, gender, and specialization of physicians, which could not be considered due to data privacy laws in Germany.  Therefore, to account for confounding by prescription habits (general as well as of the drug studied in the NIPMS) before entering into the NIPMS, we used these metrics as matching parameters. In addition, in the statistical analysis, results were adjusted for prescription practices before entering the NIPMS as well as for total prescription volume during and after the NIPMS. |
| Study size | 10 | Explain how the study size was arrived at | Methods, subsection “Sample size” | Because there are no prior studies of changes in prescription practices due to NIPMS, we had no plausible effect size estimate, and were therefore not able to calculate a sample size. We thus aimed to include all studies within the time frame with available prescription data that matched our inclusion criteria. |
| Quantitative variables | 11 | Explain how quantitative variables were handled in the analyses. If applicable, describe which groupings were chosen and why | Methods, subsection “Statistical methods”, Paragraph 1 | Mean number of package prescriptions and DDD were first calculated within each NIPMS, and then averaged across NIPMS, weighted by the number of contributing matching groups within each NIPMS. Matching-trio-wise differences were first averaged within each NIPMS, and then averaged across NIPMS, weighted by the number of contributing matching groups within each NIPMS. |
| Statistical methods | 12 | (*a*) Describe all statistical methods, including those used to control for confounding | Methods, subsection “statistical methods”, paragraphs 1-5 | Mean number of package prescriptions and DDD were first calculated within each NIPMS, and then averaged across NIPMS, weighted by the number of contributing matching trios within each NIPMS. Matching-group-wise differences were first averaged within each NIPMS, and then averaged across NIPMS, weighted by the number of contributing matching groups within each NIPMS. The weighting scheme was used to ensure that more reliable estimates based on more matching groups had more influence than more uncertain estimates based on fewer matching groups.  Conditional Poisson regression was used to assess the relative rate of prescriptions for the studied drug in participating physicians vs. their respective controls (19). Coefficients for matching groups were treated as nuisance variables and were eliminated. To account for possible overdispersion, a Quasi-Poisson approach was chosen. The log number of days of the NIPMS were used as offset. Regression models for t0 were adjusted for the total number of prescriptions during t0. Regression models for t1 and t2 were adjusted for the number of prescriptions for the studied drug during t0, and for the total number of prescriptions during t1 and t2, respectively.  Mixed binomial logistic regression was used to assess a shift in the proportion of prescriptions of studied drugs relative to alternative drugs used for the same indication in participating physicians vs. controls. The model included a random intercept effect for matching group and allowed for zero inflation since a relevant number of matching groups had 0 prescriptions for drugs under study. […]  P-values less than 0.05 were considered statistically significant. Data were analyzed using the R environment for statistical computing version 3.5.2 with packages gnm, brms, and geepack (20–23). |
|  |  | (*b*) Describe any methods used to examine subgroups and interactions | Methods, subsection “Statistical methods”, paragraph 4 | Logistic regression using generalized estimating equations was used to assess the association of marketing indicators with the probability that a participating physician prescribed more of the drug under study than the average of the corresponding controls. Clusters were defined by NIPMS with the assumption of compound symmetry for the correlation structure. |
|  |  | (*c*) Explain how missing data were addressed | Not applicable |  |
|  |  | (*d*) If applicable, explain how loss to follow-up was addressed | Not applicable |  |
|  |  | (*e*) Describe any sensitivity analyses | Methods, subsection “Sensitivity analyses”, paragraph 1 | In general, we assumed that physicians had participated in an NIPMS for the entirety of the study, even though it is likely that some physicians were recruited for participation after the NIPMS had begun or stopped participating before it was officially terminated. For a subset of physicians, a more precise time frame of participation could be inferred; this was the case when NIPMS sponsors regularly reported on the participating physicians and it was possible to determine when a specific physician first entered the study and when they stopped participating. Sensitivity analyses were conducted for this subset of physicians. In addition, sensitivity analyses were conducted that included only the drug manufactured by the sponsor of the study, as generics were available for some of the drugs studied in the NIPMS. |
| Results | | |  |  |
| Participants | 13* | (a) Report numbers of individuals at each stage of study—eg numbers potentially eligible, examined for eligibility, confirmed eligible, included in the study, completing follow-up, and analysed | Results, subsection “Study Population”, paragraph 1, S2 Figure and S1 Table | Of a total of 95 registered NIPMS that began and ended in the predefined time frame, 24 matched our inclusion criteria and 2,354 physicians that had participated in those NIPMS could be analyzed (See S1 Figure and S2 Table for information on exclusion of physicians and NIPMS, respectively). |
|  |  | (b) Give reasons for non-participation at each stage | S2 Figure and S1 Table |  |
|  |  | (c) Consider use of a flow diagram | S2 Figure |  |
| Descriptive data | 14* | (a) Give characteristics of study participants (eg demographic, clinical, social) and information on exposures and potential confounders | Results, subsection “Study Population”, paragraph 1, and S2 Table | Table 2 shows the main characteristics of included NIPMS (see S2 table for more detailed information on characteristics of the NIPMS). The mean duration of NIPMS was 500 days (SD 181d). The mean marketing score was 2.4 (SD 1.5; range 0-5, max. possible value 7.5). |
|  |  | (b) Indicate number of participants with missing data for each variable of interest | - |  |
|  |  | (c) Summarise follow-up time (eg, average and total amount) | Methods, subsection “Study design”, paragraph 1 | Defined in methods as being one year |
| Outcome data | 15* | Report numbers of outcome events or summary measures over time | Results, subsection “Primary Outcome”, paragraph 1 | Participating physicians showed consistently higher absolute prescriptions of the studied drug compared with controls, with the gap widening during t1 and narrowing during t2 (see table 3). |
| Main results | 16 | (*a*) Give unadjusted estimates and, if applicable, confounder-adjusted estimates and their precision (eg, 95% confidence interval). Make clear which confounders were adjusted for and why they were included | Results, subsection “Primary Outcome”, paragraph 1 | Relative to physicians not participating in an NIPMS, physicians participating in an NIPMS prescribed 7-8% more of the studied drug during the NIPMS and 6-7% more during the year after the NIPMS had finished, when accounting for their overall prescription volume during the respective timeframe as well as the number of prescriptions of the studied drug before the begin of the NIPMS (see table 4). |
|  |  | (*b*) Report category boundaries when continuous variables were categorized | NA |  |
|  |  | (*c*) If relevant, consider translating estimates of relative risk into absolute risk for a meaningful time period | NA |  |
| Other analyses | 17 | Report other analyses done—eg analyses of subgroups and interactions, and sensitivity analyses | Results, subsection “Primary Outcome”, paragraph 2, and S3, S4 and S5 Tables, as well as subsection “Secondary outcomes” | Sensitivity analyses of the relative prescription rates studying prescription data of all physicians regarding the studied drug taking into account only the drugs manufactured by the sponsor showed similar results, as did analyses using only data from physicians for whom a more precise time frame of participation in the NIH could be determined.; s. also Section “secondary outcomes” |
| Discussion | | |  |  |
| Key results | 18 | Summarise key results with reference to study objectives | Discussion, paragraph 1 | This study is the first to show in a quasi-experimental design that the physicians participating in an NIPMS show changed prescription rates in favor of the investigated drug. Physicians participating in a NIPMS showed a relevantly higher rate of prescription of the studied drug both during and a year after the NIPMS, with an increase of 6-8%, though the effect was slightly smaller after the NIPMS had ended. In addition, they were more likely to prescribe the studied drug rather than alternative drugs used for the same indication. This led to a trend toward a higher revenue among participating physicians, which was non-significant. None of the marketing indicators of the NIPMS proposed by our group in an earlier study were useful to predict whether an NIPMS would have a larger or smaller effect on prescription practices (9). |
| Limitations | 19 | Discuss limitations of the study, taking into account sources of potential bias or imprecision. Discuss both direction and magnitude of any potential bias | Discussion, subsection “Strengths and weaknesses”, paragraphs 2 and 3 | One strength of this study is its large sample size, studying close to 7000 physicians within a diverse collective of NIPMS. It is thus highly likely that results are generalizable to other NIPMS and other physicians. The study design was quasi-experimental, allowing for assessment of causality when certain assumptions are met. However, controlling confounders was difficult due to data privacy laws in Germany. It is unclear whether age, gender or specialization may have an influence on prescription habits. However, as mentioned in the methods section, we used directed acyclic graph modeling and to assess the effects of confounders and believe it is unlikely we insufficiently controlled for these characteristics. Only in rare cases where one of the studied drugs gains an indication during the time of the NIPMS, specialization may lead to additional confounding when one specialty would prescribe the studied drug for the new indication while another specialty would not. One other confounder that may not be sufficiently controlled for is that physicians may have chosen to participate in an NIPMS because they were already aware of a certain medication and actively wanted to gather experience using it, leading to an overestimation of the effect. In our view, however, this is not very likely because pharmaceutical companies are more likely to recruit physicians for NIPMS who are not as enthusiastic about their medication yet [27].  Another weakness is the imprecise definition of the time frame of participation in the study. Due to a lack of information regarding when exactly a physician entered or exited an NIPMS, we primarily assumed that all physicians had participated for the entire time of the NIPMS. This may lead to an under- or overestimation of the effect where we miscalculated the time frame of participation. However, our sensitivity analyses with the subset of physicians for whom a more precise time frame could be determined confirmed the effect we found in the larger set of physicians. It is thus unlikely that the effect would be changed substantially if precise data were available for all physicians. |
| Interpretation | 20 | Give a cautious overall interpretation of results considering objectives, limitations, multiplicity of analyses, results from similar studies, and other relevant evidence | Discussion, subsections “Relation to other Studies” and “Meaning and Implications”, all paragraphs | Section “Relation to other Studies” and “Meaning and Implications” |
| Generalisability | 21 | Discuss the generalisability (external validity) of the study results | Discussion, subsection “Strengths and weaknesses”, paragraph 1 | One strength of this study is its large sample size, studying close to 7000 physicians within a diverse collective of NIPMS. It is thus highly likely that results are generalizable to other NIPMS and other physicians. |
| Other information | | |  |  |
| Funding | 22 | Give the source of funding and the role of the funders for the present study and, if applicable, for the original study on which the present article is based | Section “Funding” | The authors received no specific funding for this work. |

*Give information separately for exposed and unexposed groups.
