# Supplementary material for: Impact of physicians’ participation in non-interventional post-marketing studies on their prescription habits: A retrospective 2-armed cohort study in Germany
Source: PLoS Med. 2020 Jun 26;17(6):e1003151. doi: 10.1371/journal.pmed.1003151 (PMC7319278; doi:10.1371/journal.pmed.1003151)
Supplement: S1 Table — (DOCX) [file pmed.1003151.s009.docx]

**S1 Table. Reasons for exclusions of NIPMSs**

| **Reason for exclusion** | **Number of NIPMS** |
| --- | --- |
| No prescription data for studied drug* | 20 |
| No specific drug studied | 14 |
| Included solely or mostly physicians working in hospitals | 13 |
| Retrospective study | 8 |
| Marketing authorization too late | 5 |
| Studied drug was withdrawn from the market shortly after NIPMS | 4 |
| Included dentists rather than physicians | 2 |
| No information on physicians provided by sponsor | 2 |
| No patients included in the study | 1 |
| Interventional study | 1 |
| Study was extended over the inteded time frame | 1 |
| *not subject to a prescription/ not refunded by statutory health insurance |  |
