# Supplementary material for: Impact of physicians’ participation in non-interventional post-marketing studies on their prescription habits: A retrospective 2-armed cohort study in Germany
Source: PLoS Med. 2020 Jun 26;17(6):e1003151. doi: 10.1371/journal.pmed.1003151 (PMC7319278; doi:10.1371/journal.pmed.1003151)
Supplement: S3 Table — Relative prescription rates of participating doctors versus controls considering only studied drugs manufactured by the sponsor (model for t0 adjusted for overall prescription rate; models for t1 and t2 adjusted for overall prescription rate and prescription rate of studied drug at t0). (DOCX) [file pmed.1003151.s011.docx]

**S3 Table. Sensitivity analysis 1.** Relative prescription rates of participating doctors versus controls, data for all participating physicians regarding considering only studied drugs manufactured by the sponsor (model for t0 adjusted for overall prescription rates; models for t1 and t2 adjusted for overall prescription rate and prescription rate of studied drug at t0)

|  | **Packages** | | **DDD*** | |
| --- | --- | --- | --- | --- |
|  | RR** (95% CI) | p | RR (95% CI) | p |
| t0 | 1.04 (1.03-1.05) | <0.001 | 1.04 (1.03-1.04) | <0.001 |
| t1 | 1.10 (1.08-1.12) | <0.001 | 1.09 (1.07-1.11) | <0.001 |
| t2 | 1.08 (1.06-1.10) | <0.001 | 1.06 (1.04-1.08) | <0.001 |
| * Defined daily dose of the drug studied in the NIPMS; **Relative rate; n= 2354 groups | | | | |
