# Supplementary material for: Impact of physicians’ participation in non-interventional post-marketing studies on their prescription habits: A retrospective 2-armed cohort study in Germany
Source: PLoS Med. 2020 Jun 26;17(6):e1003151. doi: 10.1371/journal.pmed.1003151 (PMC7319278; doi:10.1371/journal.pmed.1003151)
Supplement: S5 Table — Relative prescription rates of participating doctors versus controls using only data for physicians with a precise time frame of NIPMS participation and considering only drugs manufactured by the sponsor (model for t0 adjusted for overall prescription rate; models for t1 and t2 adjusted for overall prescription rate and prescription rate of studied drug at t0). (DOCX) [file pmed.1003151.s013.docx]

**S5 Table. Sensitivity Analysis 3.** Sensitivity analysis 3. Relative prescription rates of participating doctors versus controls using only data for physicians with a precise time frame of NIPMS participation and considering only drugs manufactured by the sponsor (model for t0 adjusted for overall prescription rate; models for t1 and t2 adjusted for overall prescription rate and prescription rate of studied drug at t0)

|  | **Packages** | | **DDD*** | |
| --- | --- | --- | --- | --- |
|  | RR** (95% CI) | P | RR (95% CI) | p |
| t0 | 1.05 (1.03-1.06) | <0.001 | 1.06 (1.04-1.07) | <0.001 |
| t1 | 1.10 (1.07-1.12) | <0.001 | 1.08 (1.06-1.11) | <0.001 |
| t2 | 1.03 (1.00-1.06) | 0.04 | 1.01 (0.98-1.04) | 0.5 |
| * Defined daily dose of the drug studied in the NIPMS; **Relative rate; n= 1,286 groups | | | | |
