# Supplementary material for: Impact of physicians’ participation in non-interventional post-marketing studies on their prescription habits: A retrospective 2-armed cohort study in Germany
Source: PLoS Med. 2020 Jun 26;17(6):e1003151. doi: 10.1371/journal.pmed.1003151 (PMC7319278; doi:10.1371/journal.pmed.1003151)
Supplement: S6 Table — Note that only alternative drugs that shared the first 3 places of the ATC code with the studied drug were included. For some studied drugs, alternative medications with a different ATC code may exist. In addition, for some alternative drugs, the dosage form was restricted to ensure comparability. (DOCX) [file pmed.1003151.s014.docx]

**S6 Table. Alternative Drugs for each NIMPS**. Note that only alternative drugs that shared the first three places of the ATC-code with the studied drug were included. For some studied drugs, alternative medications with a different ATC-code may exist. In addition, for some alternative drugs, the dosage form was restricted to ensure comparability.

| **NIPMS** | **Studied drug** | **Indication** | **Alternative drug(s)** |
| --- | --- | --- | --- |
| 1 | S01BA15 (Floucinolon acetonide) | Diabetic macula edema | S01BA01 (Dexamethasone)  *(only implants)* |
| 2 | D07AC13 (Mometasone) | Psoriasis | D07AA01 (Methylprednisolone)  D07AA02 (Hydrocortisone)  D07AA03 (Prednisolone)  D07AB02 (Hydrocortisone butyrate)  D07AB03 (Flumetasone)  D07AB09 (Triamcinolone)  D07AB11 (Hydrocortisone buteprate)  D07AB19 (Dexamethasone)  D07AB21 (Clocortolone)  D07AC01 (Betametasone)  D07AC02 (Fluclorolone)  D07AC03 (Desoximetasone)  D07AC04 (Fluocinolone acetonide)  D07AC05 (Fluocortolone)  D07AC08 (Fluocinonide)  D07AC11 (Amcinonide)  D07AD01 (Clobetasole)  D07AD02 (Halcinonide)  D07AB07 (Fluprednidene)  D07AC06 (Diflucortolone)  D07AC10 (Diflorasone)  D07AC14 (Methylprednisolone aceponate)  D07AC16 (Hydrocortisone aceponate)  D07AC17 (Fluticasone)  D07AC18 (Prednicarbate)  *(only transdermal dosage forms)* |
| 3 | L01CD01 (Paclitaxel) | Breast cancer, non-small cell lung cancer, ovarian cancer | L01CA01 (Vinblastine)  L01CA02 (Vincristine)  L01CA04 (Vinorelbine)  L01CB01 (Etoposide)  L01CD04 (Cabazitaxel)  L01CD02 (Docitaxel) |
| 4 | J05AP02 (Telaprevir) | Hepatitis C | J05AP01 (Ribavirin)  J05AP03 (Boceprevir)  J05AP04 (Faldaprevir)  J05AP05 (Simeprevir)  J05AP06 (Asunaprevir)  J05AP07 (Daclatasvir)  J05AP08 (Sofosbuvir)  J05AP09 (Dasabuvir)  J05AP51 (Sofosbuvir and Ledipasvir)  J05AP52 (Dasabuvir, Ombitasvir, Paritaprevir and Ritonavir)  J05AP53 (Ombitasvir, Paritaprevir and Ritonavir)  J05AP54 (Elbasvir and Grazoprevir)  J05AP55 (Sofosbuvir and Velpatasvir)  J05AP56 (Sofosbuvir, Velpatasvir and Voxilaprevir)  J05AP57 (Glecaprevir and Pibrentasvir) |
| 5 | L01XE05 (Sorafenib) | Hepatocellular carcinoma | None |
| 6 | L03AA02 (Filgrastim)/  L03AA13 (Pegfilgrastim) | Neutropenia | L03AA10 (Lenograstim)  L03AA14 (Lipegfilgrastim)  L03AA16 (Empegfilgrastim)  L03AA09 (Sargramostim)  L03AA15 (Balugrastim) |
| 7 | L04AB02/  L04AB06 (Infliximab/ golumimumab) | Ankylosing spondilitis | L04AB01 (Etanercept)  L04AB04 (Adalimumab)  L04AB05 (Certolizumab pegol)  L04AC10 (Secukinumab)  L04AA32 (Apremilast) |
| 8 | N02AX06 (tapentadol) | Low back pain | N02AA01 (Morphine)  N02AA03 (Hydromorphone)  N02AA05 (Oxycodone)  N02AA08 (Dihydrocodeine)  N02AA55 (Oxycodone and naloxone)  N02AB02 (Pethidine)  N02AB03 (Fentanyl)  N02AC03 (Piritramide)  N02AE01 (Buprenorphine)  N02AX02 (Tramadol)  N02AX51 (Tilidine and naloxone)  N02AJ06 (Codeine and paracetamol)  N02AJ07 (Codeine and acetylsalicylic acid)  N02AJ08 (Codeine and ibuprofen)  N02AJ09 (Codeine and other non-opioid analgesics)  N02AJ13 (Tramadol and paracetamol)  N02AJ14 (Tramadol and dexketoprofen)  N02AJ15 (Tramadol and other non-opioid analgesics)  N02AJ17 (Oxycodone and paracetamol)  N02AJ18 (Oxycodone and acetylsalicylic acid)  N02AJ19 (Oxycodone and ibuprofen)  N02AX01 (Tilidine)  N02AJ05 (Codeine and diclofenac)  N02AJ03 (Dihydrocodeine and other non-opioid analgesics)  N02AJ02 (Dihydrocodeine and acetylsalicylic acid)  N02AJ01 (Dihydrocodeine and paracetamol)  *(only oral and transdermal dosage forms)* |
| 9 | B03XA02 (Darbepoetin alfa) | Anemia | B03XA01 (Erythropoietin)  B03XA03 (Methoxy-Polyethylenglycol-Epoetin beta)  B03XA05 (Epoetin delta) |
| 10 | L01CD02 (Docetaxel) | Breast cancer, non-small cell lung cancer, prostate cancer, adenocarcinoma of the stomach, squamous cell carcinoma of the head/neck region | L01CA01 (Vinblastine)  L01CA02 (Vincristine)  L01CA04 (Vinorelbine)  L01CB01 (Etoposide)  L01CD04 (Cabazitaxel)  L01CD01 (Paclitaxel) |
| 11 | M05BX04 (Denosumab) | Osseous metastatic disease | M05BA02 (Clodronic acid)  M05BA03 (Pamidronic acid)  M05BA04 (Alendronic acid)  M05BA06 (Ibandronic acid)  M05BA08 (Zoledronic acid)  M05BA07 (Risedronic acid)  M05BA01 (Etidronic acid)  M05BA04 (Alendronic acid) |
| 12 | L04AB02 (Infliximab) | Rheumatoid arthritis | L04AA13 (Leflunomide)  L04AA24 (Abatacept)  L04AA29 (Tofacitinib)  L04AA37 (Baricitinib)  L04AB01 (Etanercept)  L04AB04 (Adalimumab)  L04AB05 (Certolizumab pegol)  L04AC03 (Anakinra)  L04AC07 (Tocilizumab)  L04AC14 (Sarilumab)  L04AD01 (Ciclosporin)  L04AX01 (Azathioprine)  L04AX03 (Methotrexate)  L04AB02 (Infliximab)  L04AB06 (Golimumab) |
| 13 | L04AD01 (Ciclosporin) | Psoriasis | L04AA21 (Efalizumab)  L04AA32 (Apremilast)  L04AB01 (Etanercept)  L04AB02 (Infliximab)  L04AB04 (Adalimumab)  L04AC05 (Ustekinumab)  L04AC10 (Secukinumab)  L04AC12 (Brodalumab)  L04AC13 (Ixekizumab)  L04AX01 (Azathioprine)  L04AX03 (Methotrexate) |
| 14 | B03AC06 (Iron (III) isomaltoside) | Anemia | B03AA01 (Iron(II) glycinsulfate)  B03AA03 (Iron(II) gluconate)  B03AA06 (Iron(II) succinate)  B03AA07 (Iron(II) sulfate)  B03AC01 (Iron(III)-hydroxide polymaltose complex)  B03AC02 (Iron(III)-oxide saccharose complex)  B03AC03 (Iron(III)-sorbitol citric acid complex)  B03AC05 (Iron(III)-sorbitol gluconic acid complex)  B03AC07 (Iron(III)-sodium gluconate complex)  B03AA50 (Combinations of iron(II) and iron(III))  B03AA20 (Combinations)  B03AA13 (Iron(II)polystyrene sulfonate)  B03AA12 (Ammonium iron(II) sulfate)  B03AA11 (Ferrous iodine)  B03AA10 (Ferrous ascorbate)  B03AA02 (Ferrous fumarate)  B03AA04 (Ferrous carbonate)  B03AA05 (Ferrous chloride)  B03AA08 (Ferrous tartrate)  B03AA09 (Ferrous aspartate)  B03AB05 (Ferric oxide polymaltose complexes) |
| 15 | N04BD02 (Rasagiline) | Parkinson’s disease | N04BA01 (Levodopa)  N04BA03 (Levodopa, decarboxylase inhibitor and COMT inhibitor)  N04BA10 (Levodopa in combination with carbidopa)  N04BA11 (Levodopa in combination with benserazide)  N04BA13 (Levodopa in combination with carbidopa und entacapone)  N04BA14 (Levodopa in combination with carbidopa und pramipexole)  N04BB01 (Amantadine)  N04BC01 (Bromocriptine)  N04BC04 (Ropinirole)  N04BC02 (Pergolide)  N04BC03 (Dihydroergocryptine mesylate)  N04BC05 (Pramipexole)  N04BC06 (Cabergoline)  N04BC08 (Piribedil)  N04BC09 (Rotigotine)  N04BC10 (Lisuride)  N04BD01 (Selegiline)  N04BD03 (Safinamide) |
| 16 | N06DA03 (Rivastigmine) | Dementia | N06DA02 (Donepezil)  N06DA04 (Galantamine)  N06DX01 (Memantine)  N06DX13 (Nicergoline)  N06DX19 (Dihydroergocristine)  N06DX18 (Nimodipine) |
| 17 | D11AF55 (Fluorouracil and salicylic acid) | Actinic keratosis | D11AX18 (Diclofenac)  D11AF05 (Fluorouracil) |
| 18 | G04BD06 (Propiverine) | Overactive bladder | G04BD02 (Flavoxate)  G04BD04 (Oxybutynin)  G04BD07 (Tolterodine)  G04BD08 (Solifenacin)  G04BD09 (Trospium)  G04BD10 (Darifenacin)  G04BD11 (Fesoterodine)  G04BD12 (Mirabegron)  G04BP01 (Bearberry leaves)  G04BP06 (Solidago)  G04BP07 (Pumpkin seeds) |
| 19 | D06BX02 (Ingenol mebutate) | Actinic keratosis | D06BB10 (Imiquimod) |
| 20 | N06AX22 (Agomelatine) | Depression | N06AA01 (Desipramine)  N06AA02 (Imipramine)  N06AA03 (Imipramine oxide)  N06AA04 (Clomipramine)  N06AA06 (Trimipramine)  N06AA07 (Lofepramine)  N06AA08 (Dibenzepin)  N06AA09 (Amitriptyline)  N06AA10 (Nortriptyline)  N06AA12 (Doxepin)  N06AA13 (Iprindole)  N06AA16 (Dosulepin)  N06AA17 (Amoxapine)  N06AA21 (Maprotiline)  N06AB03 (Fluoxetine)  N06AB04 (Citalopram)  N06AB05 (Paroxetine)  N06AB06 (Sertraline)  N06AB08 (Fluvoxamine)  N06AB10 (Escitalopram)  N06AF01 (Isocarboxazid)  N06AF03 (Phenelzine)  N06AF04 (Tranylcypromine)  N06AG02 (Moclobemide)  N06AP01 (Hypericum)  N06AX03 (Mianserin)  N06AX05 (Trazodone)  N06AX11 (Mirtazapine)  N06AX12 (Bupropion)  N06AX14 (Tianeptine)  N06AX16 (Venlafaxine)  N06AX17 (Milnacipran)  N06AX18 (Reboxetine)  N06AX21 (Duloxetine)  N06AX23 (Desvenlafaxine)  N06AX26 (Vortioxetine) |
| 21 | G03BA03 (Testosterone) | None specified | G03BB02 (Androstanolone)  G03BA02 (Methyltestosterone) |
| 22 | S01ED62 (Timolol and bimatoprost) | Glaucoma | S01EA02 (Dipivefrine)  S01EA03 (Apraclonidine)  S01EA04 (Clonidine)  S01EA05 (Brimonidine)  S01EA52 (Dipivefrine, combinations)  S01EB01 (Pilocarpine)  S01EB02 (Carbachol)  S01EB04 (Demecarium)  S01EB05 (Physostigmine)  S01EB06 (Neostigmine)  S01EC01 (Acetazolamide)  S01EC02 (Diclofenamide)  S01EC03 (Dorzolamide)  S01EC04 (Brinzolamide)  S01EC05 (Methazolamide)  S01EC24 (Brinzolamide and brimonidine)  S01EC54 (Brinzolamide, combinations)  S01ED01 (Timolol)  S01ED02 (Betaxolol)  S01ED03 (Levobunolol)  S01ED04 (Metipranolol)  S01ED05 (Carteolol)  S01ED08 (Bupranolol)  S01ED24 (Metipranolol and pilocarpine)  S01ED51 (Timolol, combinations)  S01ED52 (Betaxolol, combinations)  S01ED54 (Metipranolol, combinations)  S01ED55 (Carteolol, combinations)  S01ED61 (Timolol and latanoprost)  S01ED63 (Timolol and travoprost)  S01ED66 (Timolol and dorzolamide)  S01ED67 (Timolol and brinzolamide)  S01ED68 (Timolol and pilocarpine)  S01ED69 (Timolol and brimonidine)  S01ED70 (Timolol and tafluprost)  S01EE01 (Latanoprost)  S01EE02 (Unoprostone)  S01EE03 (Bimatoprost)  S01EE04 (Travoprost)  S01EE05 (Tafluprost)  S01EB21 (Pilocarpine and phenylephrine)  S01ED07 (Pindolol) |
| 23 | C01EB17 (Ivabradine) | Chronic heart failure | None |
| 24 | R03AC19/ R03BB04 (Olodaterol/ Tiotropium bromide | COPD | R03AC03 (Terbutaline)  R03AC04 (Fenoterol)  R03AC13 (Formoterol)  R03AC18 (Indacaterol)  R03AK10 (Vilanterol and fluticasone furoate)  R03AL01 (Fenoterol and ipratropium bromide)  R03AL03 (Vilanterol and umeclidinium bromide) R03AL04 (Indacaterol and glycopyrronium bromide) R03AL06 (Olodaterol and Tiotropium bromide)  R03AC12 (Salmeterol)  R03AK06 (Salmeterol and fluticasone)  R03AK07 (Formoterol and budesonide)  R03AK08 (Formoterol and beclometasone)  R03AK09 (Formoterol and mometasone)  R03AK11 (Formoterol and fluticasone)  R03AK12 (Salmeterol and budesonide)  R03AL05 (Formoterol and aclidinium bromide)  R03AL07 (Formoterol and glycopyrronium bromide)  R03AL09 (Formoterol, glycopyrronium bromide and beclometasone)  R03BA01 (Beclometasone)  R03BB01 (Ipratropium bromide)  R03BB05 (Aclidinium bromide)  R03BB06 (Glycopyrronium bromide)  R03BB07 (Umeclidinium bromide)  R03BA05 (Fluticasone)  R03BA02 (Budesonide)  R03BB54 (Tiotropium bromide, combinations)  R03BB02 (Oxitropium bromide)  *(only inhalable dosage forms)* |
