# Supplementary material for: Impact of physicians’ participation in non-interventional post-marketing studies on their prescription habits: A retrospective 2-armed cohort study in Germany
Source: PLoS Med. 2020 Jun 26;17(6):e1003151. doi: 10.1371/journal.pmed.1003151 (PMC7319278; doi:10.1371/journal.pmed.1003151)
Supplement: Attachment — Submitted filename: Response_to_reviewers.docx [file pmed.1003151.s015.docx]

**Response to the comments by the editors and reviewers regarding the manuscript “Effect of physicians’ participation in non-interventional post-marketing studies on their prescription habits: A retrospective two-armed cohort study”**

**Editors’ requests**

*Abstract – Please add dates of the study as well as geographic information. I would usually ask for summary demographic information to be added to the abstract and it isn’t really fully relevant here, but I still think some information like average age and sex ratio would be good. Also Please be more explicit about what the study’s limitations are as the final sentence of the ‘Methods and Findings’ section.*

We have added the dates of the study and it was already mentioned in the abstract that this was a study in Germany. However, we don’t have information regarding age and sex of the physicians. We have added a sentence on the limitations of the study in the “Methods and Findings” section as follows: *“The main limitation was the difficulty in controlling for confounders due to privacy laws with a resulting lack of information regarding the included physicians, which was mainly addressed by the matching process.”*

*At this stage, we ask that you include a short, non-technical Author Summary of your research to make findings accessible to a wide audience that includes both scientists and non-scientists. The Author Summary should immediately follow the Abstract in your revised manuscript. This text is subject to editorial change and should be distinct from the scientific abstract. Please see our author guidelines for more information:* [*https://journals.plos.org/plosmedicine/s/revising-your-manuscript#loc-author-summary*](https://journals.plos.org/plosmedicine/s/revising-your-manuscript#loc-author-summary)

We have added an author summary as requested.

*Data – even though data can’t be shared by you, please provide the email or link where you requested the data from so others can do the same.*

As we mention in the manuscript, the acquisition of data was a complicated process that required getting permission from the Ministry of Health and months of data collection at two locations. We have mentioned all the actors that are in possession of the data, but it is not possible to just name a link or e-mail from which to request the data set. However, we have offered to send another request to the Ministry of Health to allow us to publish our anonymized final data set, if desired. We cannot guarantee for the outcome of such a request, however.

*Refs in main text - Please use square brackets instead of rounded.*

*Formatting errors/ - Line 99, for example. There appear to be errors in the main text )Error! Bookmark not defined..*

We have changed the brackets to square and have gone over the paper again regarding the bookmark errors. These should not appear any more.

*Did your study have a prospective protocol or analysis plan? Please state this (either way) early in the Methods section.*

*a) If a prospective analysis plan (from your funding proposal, IRB or other ethics committee submission, study protocol, or other planning document written before analyzing the data) was used in designing the study, please include the relevant prospectively written document with your revised manuscript as a Supporting Information file to be published alongside your study, and cite it in the Methods section. A legend for this file should be included at the end of your manuscript.*

*b) If no such document exists, please make sure that the Methods section transparently describes when analyses were planned, and when/why any data-driven changes to analyses took place.*

*c) In either case, changes in the analysis-- including those made in response to peer review comments-- should be identified as such in the Methods section of the paper, with rationale.*

Our analysis plan was based on the request we submitted to the Ministry of Health, which we have now submitted as additional supporting information. This file is in German, please let us no if we should translate the document. No data-driven changes in the analysis were made and no other changes in the analysis were made in response to peer review.

*Please complete the CONSORT checklist and ensure that all components of CONSORT are present in the manuscript, including [how randomization was performed, allocation concealment, blinding of intervention, definition of lost to follow-up, power statement].*

As previously discussed via e-mail, the STROBE statement seems to be more appropriate for our study, so we have submitted this statement again with the revised manuscript.

**Reviewer #1**

*The authors report the findings of a quasi-experimental study evaluating the effects of non-interventional post-marketing studies on prescribing volume rate of prescribing of the particular drug studied in the NIPM during and in the following year. They matched every intervention physician with 2 controls who were not participants in an NIPM study.
The study found that the relative rate of prescribing of physicians in NIPM studies were higher for particular drug studied during and in the year after.

The strengths of the study include its large study sample, attempt to control for confounding through matching by prescription volume of prescriptions and defined daily dosage in the year before, and the use of DAGs to conceptualise the potential causal pathways.

The use of a conditional Poisson regression analysis to determine the relative rates is appropriate type of statistical model for this type of analysis with an outcome related to count data.*

*Major comments

The primary weaknesses are due to its non-randomised design, only matching based on a very limited number of factors (due to availability of data due to privacy laws), confounding by indication and residual confounding (e.g. propensity for certain characteristics of physicians who may join a NIPM study in the first place are not taken into account), lack of a sample size calculation on a potential hypothesized effect size.

The authors could have also considered, for the experimental arm (physicians involved in NIPMS), a negative exposure drug which was the studies drug under question and conduct a sensitivity analyses to look at the relative rate of a non-studied drug compared to matched controls. Critically, if there still is an effect, it is likely that there will be residual confounders which were not considered in the primary analyses. If there is no effect - this would strengthen the conclusion of the paper. If there is an effect - unfortunately this would make their findings less than definitive.

Due to this - my feeling was the conclusion was too definitive given the limitations in the non-randomised design of the study as well as the short-time horizon (for instance, does this effect persist after the NIPM ends: the results in Table 3 show that at t1 the prescribing increases but then substantially falls towards t2 at 1 year). The authors' primary conclusion that NIPMS should be more strictly regulated due to changes in prescribing practices in favour of the investigated drug. However, the key issue that hasn't been investigated is whether any of these changes prescribing practices actually result in clinical benefit, clinical harm, or no change for the patient.*

We agree that a randomized design would be the ideal design to test for a causal effect such as the one we are looking for; however, this is obviously not feasible in this case. Thus, we have turned to the next-best design under the circumstances.

Regarding the consideration of a negative exposure drug, we agree that the lack of any effect for NIPMS-group vs. control-group would indeed lend better validity to the observed results. However, such a group of control drugs would have to firstly be completely uncorrelated with the NIPMS drug itself as well as related drugs and secondly be chosen taking into account the specializations of the physicians, such that a-priori, they would have been equally likely to be prescribed. Since we are not aware of the specializations of the physicians, choosing the control drugs was not possible. Therefore, while appealing, we have no means to carry out this analysis.

With respect to self-selection effects from voluntary NIPMS participation / “confounding by indication” bias, we can be reasonably sure to have eliminated confounding due to prior prescription behavior with respect to NIPMS drugs, and with respect to total prescription volume, with our matching process. We have assessed the matching quality with respect to these two criteria. There was an average relative difference of 0.2% for overall packages and -0.83% for overall DDD between cases and controls during time period t0. There was an average relative difference of 8.29% for number of packages and 10.13% for number of DDD of the studied drug between cases and controls during time period t0. To account for remaining differences in prescription volume despite matching, Poisson regression models for time period t1 adjusted for the number of NIPMS like drugs prescriptions during t0, and for the total number of prescriptions during t1. We would therefore argue that the observed higher relative prescription volume at t1 of NIPMS participants cannot be attributed to self-selection effects with respect to NIPMS participation before t1 in the sense of confounding by indication. Still, we mention in our limitations that there is the rather unlikely possibility that physicians already interested in a specific drug will be more likely to participated in an NIPMS. This seems unlikely because pharmaceutical companies are more likely to target physicians that are not already prescribing the drug (cf. lines 395 ff).

At first glance, it might seem to make sense to use the trial by Andersen et al. mentioned in one of the comments below to estimate a sample size. However, this trial studied the effect of a randomized trial on physicians’ prescribing behavior. Such a trial has a protocol directly dictating prescription behavior, so that one would expect a more pronounced effect on physicians’ prescription practices than from the specifically non-interventional post-marketing studies we studied. Using this effect size could thus have led to an underpowered study for our objective.

Finally, it is true that our study was not able to assess whether the change in physicians’ prescription behavior is clinically relevant to patient outcomes. Possibly our conclusion was too strongly worded in light of this fact. However, we stand by the point that there is overwhelming evidence that most NIPMS are not scientifically sound and do not result in positive outcomes for patients due to rarely being published, as we mention in the introduction of the manuscript. With the additional risk that they may change physicians’ prescription practices, they should only be allowed when their design allows for the collection of meaningful data and thus a benefit that balances the possible harms.

*Minor comments:

Line 99 - check reference link, seems to be an error*

We have checked the document and errors such as these should no longer occur.

*Sample size calculation: There must be some rationale for 1:2 matching or else why not just run the analysis on 1:1 matching.  In the discussion, the authors mention a previous trial (ref 15) Anderson et al. which did seem to have a measure of increase % of sales in an interventional trial. Could the authors not have used the trials figures to give an estimated samples size powered on an expected difference from the cited trial?*

Thank you for the opportunity to clarify our rationale for matching. 1:2 matching of cases to controls was chosen as a balance between increasing statistical power through increasing the number of controls [1,2], and matching quality given that the pool of good matches for each case was limited. In general, increasing the number of controls per case from 1 to 2 leads to the highest relative increase in statistical power while adding more than 2 controls has diminishing returns. We have added this rationale in the manuscript: *“Two controls were chosen to balance the increase in statistical power through the number of controls with the matching quality given the limited pool of good matches for each case.”* (lines 191 ff).

We have answered the question regarding the sample size calculation in the reply to the reviewer’s previous major comment. Due to the difference in trial design, a sample size calculation from the mentioned study by Andersen et al. would not have yielded a useful result.

[1] Hennessy S, Bilker WB, Berlin JA, Strom BL. Factors Influencing the Optimal Control-to-Case Ratio in Matched Case-Control Studies. American Journal of Epidemiology 1999; 149(2): 195-197.

[2] Wacholder S, Silverman DT, McLaughlin JK, Mandel JS. Selection of Controls in Case-Control Studies: III. Design Options. American Journal of Epidemiology 1992; 135(9): 1042-1050.

*Table 3 results - Could the authors explain why there an increase for both control and NIPMS groups during t1 and the residual effects seems to reduce over time for both groups*

We understand why this might be confusing. It was not clearly stated that while t0 and t2 were of the same length in all NIPMS (1 year), t1 was the duration of the NIPMS, i.e. often a duration of more than one year. The absolute prescriptions during this time are therefore higher in both groups simply due to the longer time period. This is now mentioned directly in the legend of table 3: *Note that t1 is not the same duration as t0 and t2; number of prescriptions may be compared between groups during the same time frame, but not between time frames.*

*Line 341 - Statement is factual incorrect - The study itself was not a trial design and quasi-experimental trials are not designed to specifically assess causality but rather this type design allows you to use casual inference methods when RCT are infeasible.*

Indeed the word “trial” was not used correctly in this sentence, thank you for pointing this out. We have also somewhat weakened the rest of the statement by stating that certain assumptions have to be met to allow for causal inference methods. ”*The study design was quasi-experimental, allowing for assessment of causality when certain assumptions are met.” (lines 385 f).*

*Lines 356 - Authors touch on this issue in the discussion, but how you deal confounding by indication that those physicians who also participate in NIPMs are more likely to sign up to medications may be interested in or have a preference for? One suggestion could be to consider a negative control drug (which is not studied in the NIPMS) for participating physician.*

As we mentioned in our above reply to the major comments, we agree that such a negative control drug would be a worthwhile analysis, however, we are not able to carry out this analysis with the data we have.

**Reviewer #2**

*This is a fascinating article written on a highly important subject. The influence of industry in medical practice is of great concern globally. One particular way in which researchers have suspected that industry might try to influence physicians is through the conduct of post-marketing trials, which are ostensibly about generating new scientific information but are hypothesized to serve primarily marketing or "seeding" purposes. However, due to lack of uniform data across different companies, trials, and health care institutions, this hypothesis has been difficult to test. The authors of this study are to be commended for their application of a novel data source which appears to be exactly the kind of data needed to test this hypothesis.

I have several suggestions below to improve the manuscript. I will divide these into "major" and "minor" comments; in my view "major" comments are those that I would consider mandatory to be adequately addressed before publication. "Minor" comments are recommended, but may not be essential.*

We thank the reviewer for this positive assessment of the importance of our article and the methods we used. We are happy to reply to the further comments below.

*Major recommendations:
1. The manuscript is not clearly written. It would greatly benefit from the assistance of a medical writer to help edit for flow, style, and clarity. There are also several places in the manuscript, particularly the Methods, which need to be reorganized in order to convey more clearly what analyses were done. 2. Related to comment (1) above, I am still not 100% sure that I have fully understood the analytic approach that was taken in this paper. The analysis appears to be strong, but I would want to review the methodology again after it has been edited for clarity in order to make sure that this is the case.*

In response to these two comments by this reviewer, we have reviewed the manuscript and edited for clarity where necessary. We are confident that the analysis should now be sufficiently clearly described.

*3. Line 127 refers to "eligible NIMPS," which as of this point has not been defined yet. I would suggest starting the methods section with a full and clear description of inclusion of NIMPS before launching in to other details. This is in line with comment #1 above regarding need to reorganize the Methods section for greater clarity.*

We appreciate the specific suggestion for a change in organization. However, we believe that it is helpful to readers to first gain an overview of the study design before launching into details such as the exact eligibility criteria of the NIPMS. We have now added a reference to the respective table earlier in the methods section for those readers that want to skip ahead to the eligibility criteria.

*4. The analysis of prescribing during t1 needs to be described more fully. Periods t0 and t2 are both presented as being exactly 1 year in length. But t1 is not, and will be different for every NIMPS. Were prescriptions during t1 annualized? If so, did the authors account for uncertainty in estimates arising from shorter NIMPS being "inflated" up to a full year period?*

We are grateful for the opportunity to clarify this point. The descriptive measures in Table 3 were calculated as weighted averages across individual NIPMS with weights equal to the number of matching groups in a NIPMS. Each matching group therefore had the same weight in the averaged value irrespective of length of NIPMS. This means that in table 3, prescription volume is only comparable between groups, not within groups. We have added a legend to the table to clarify as follows: “*Note that t1 is not the same duration as t0 and t2; number of prescriptions may be compared between groups during the same time frame, but not between time frames*.”

In the conditional Poisson regression model used to analyze the association of group membership (NIPMS vs. control) with prescription behavior, the NIPMS duration was taken into account when evaluating the absolute number of prescribed packages. Implicitly, the model analyzes the number of prescriptions per unit of time. To account for possible overdispersion, a Quasi-Poisson approach was chosen. This allows for different uncertainties associated with different NIPMS. To clarify, the manuscript has been adjusted slightly: *“To account for possible overdispersion, a Quasi-Poisson approach was chosen. The log number of days of the NIPMS were used as offset.” (lines 260 f)*

*5. Related to #4 above, the authors refer to prescribing "rates," but the results I see appear to be simply prescribing counts. A rate implies a "per unit time" in the measure. Are the results presented in Table 3 (for example) the number of prescriptions PER YEAR? If so, this would in fact be a rate…but this should be described more clearly.*

We apologize that this was not sufficiently clear. In some instances, such as the descriptive analysis with results presented in Table 3, absolute numbers of prescriptions were used. In this case, it is indeed not a rate but an absolute number of packages that is presented.

However, the Poisson regression model considered prescription rates (packages/DDD per unit of time) by including the log number of days as an offset in the model, which is why we refer to rates in this case. As mentioned above, we have tried to clarify in the description of statistical methods.

*6. More detail needs to be given regarding the "indicators of marketing." Because of the centrality of these measures to the current study, I don't think it is enough to refer only to the prior publication. It would be fine to put it in the supplement, but I think more description (are these measures objective? Subjective? What criteria were they based on?) needs to be present in the current manuscript.*

We are happy to offer more detail on the marketing indicators and have now included these in the S4 Appendix.

*7. The authors need to include results regarding the success of their matching algorithm. The central question that needs to be addressed here is how closely the exposed and control physicians were able to be matched on their prescription counts. For example, the difference in the number of prescriptions between the exposed and control physicians could be determined within each matched trio, and then the distribution of the difference across all matched trios could be shown in a supplementary table. Without presentation of data such as these (or some other analysis to the same effect), the apparent difference between exposed and control physicians in terms of drug prescribed during the t0 period is highly concerning.*

We have assessed the matching quality with respect the overall packages and DDD prescribed as well as the packages and DDD of the studied drug prescribed. There was an average relative difference of 0.2% for overall packages and -0.83% for overall DDD between cases and controls during time period t0. There was an average relative difference of 8.29% for number of packages and 10.13% for number of DDD of the studied drug between cases and controls during time period t0. We have added this information in the results under “Matching quality” (lines 298 ff). To account for remaining differences in prescription volume despite matching, Poisson regression models for time period t1 adjusted for the number of NIPMS like drugs prescriptions during t0, and for the total number of prescriptions during t1. We would therefore argue that the observed higher relative prescription volume at t1 of NIPMS participants cannot be attributed to self-selection effects with respect to NIPMS participation before t1 in the sense of confounding by indication. Still, we mention in our limitations that there is the rather unlikely possibility that physicians already interested in a specific drug will be more likely to participated in an NIPMS. This seems unlikely because pharmaceutical companies are more likely to target physicians that are not already prescribing the drug (cf. lines 395 ff).

*8. I believe that physician specialty has a potential role as a confounder beyond that which is discussed by the authors. For some drugs, physician specialty could affect prescriptions at t1 independent of the effect at t0; this is because drugs are constantly gaining new indications and usages which may apply to only some specialties. For example, infliximab has longstanding uses for rheumatologic/inflammatory conditions, but increasing use in treating immunotherapy-related toxicity in cancer patients. Therefore, I would expect t0 = t1 for a rheumatologist or gastroenterologist, but t1 > t0 for an oncologist. The authors state that physician specialty is not available to them, so I think that specialty needs more discussion in Limitations as an unmeasured confounder.*

Thank you for pointing out this possible additional role of specialization as a confounder. We don’t believe that it will have a significant impact on the results of our study because a relevant number of drugs would have to have gained such new indications during the exact time frame of the NIPMS while specializations would have to be unevenly distributed between groups. We believe this is rather unlikely. However, we have added a discussion of this point to the limitations as follows: *“Only in rare cases where one of the studied drugs gains an indication during the time of the NIPMS specialization may lead to additional confounding when one specialty would prescribe the studied drug for the new indication while another specialty would not.”* (lines 390 ff).

*9. My best understanding of the results is that lines 220-225 describe the analysis presented in table 4. However, lines 226-229 appear to describe a separate analysis, and it is not clear to me where these results are presented?*

We are happy to clarify: The reviewer has correctly identified the first analysis. The second analysis refers to the change in proportion of studied drug compared with the alternative drugs used for the same indication and is presented under “Shift in Prescription practices and financial effect”. To make the identification easier, we have adjusted the manuscript as follows. Line 327, subtitle has been changed to “*Shift in Proportion of studied drugs and financial effect*”, and line 329 “*drug used for the same indication*” has been added.

*Minor recommendations:
1. Abstract, line 49 (and other locations): the comparison of different exposure groups is implicit within retrospective cohort studies. Therefore, the term "two arm" is not needed, and confusedly may be taken to imply that this was a randomized study.*

While we agree that for the most part, cohort studies are done for comparison of two different exposure groups, there are some single-armed retrospective cohort studies (i.e. this one: <https://www.ncbi.nlm.nih.gov/pubmed/29417268> ), so that in our opinion it does make sense to state that our study compared two arms. We also believe that it is unlikely to be confused with a randomized study as we explicitly state that it is a cohort study.

*2. Line 80: What does "pharmaceutical authorities" refer to? Drug companies? Regulatory bodies such as the FDA and the EMA? If the latter, "regulatory agencies" might be a better term.*

Thank you for pointing out this error in terminology; indeed we were referring to agencies such as the FDA and the EMA and the equivalent in Germany. The term has been changed to “regulatory agencies” (line 110).

*3. Line 90: the phrase "the fact that NIPMS seem to be published extremely rarely" does not make sense. See major recommendation #1 above regarding need for style and clarity edits.*

We have removed this phrase to clarify. The sentence now reads: *“NIPMS are also published extremely rarely, which makes it even less likely that they will lead to safer precription practices, even if relevant data is generated.“ (line 119 ff).*

*4. Line 99: "Error, bookmark not defined"*

We have checked the document and errors such as these should no longer occur.

*5. Lines 213-14: Authors state that trios without "at least one control" were excluded. Does this mean that those with only one control were still included? These would be pairs, not trios. Hence, "trio" might not be the best term if some of them were in fact 1:1 rather than 1:2.*

Indeed, the matching “trios” with only one control were included, so that we have decided on “matching group” as a more accurate term than “trio”, referring to either pairs or trios.

*6. Line 221: Explain further. How were coefficients for matching trios generated? Was this a fixed-effects model, with trio-level effects?*

The reviewer is correct in that in principle, the model treats matching group as a fixed effect like an ordinary grouping factor with one parameter per matching group (except for the reference group). However, in conditional Poisson regression, these parameters need not actually be estimated, and hence no degrees of freedom are spent on these parameters [1].

[1] Armstrong BG, Gasparrini A, Tobias A. Conditional Poisson models: a flexible alternative to conditional logistic case cross-over analysis. BMC Medical Research Methodology 2014; 122.

*7. Table 3. How do the authors interpret the apparent increase in prescriptions among controls during t1? It does not seem to me that prescribing should increase at all in this group.*

This effect is due to the fact that t1 is (on average) longer than t0 and t2, leading to higher absolute prescription numbers. This has been addressed by our answer to the previous major comment 4.

*8. Line 288: Does this statistic (1.04 with 95% CI 1.03-1.05) refer back to the first row of table 4? If so, why is this statistic described as an Rpr in one place and an OR in the other? If not, what table does it refer to?*

It is understandable that due to the exact same numbers, one could suspect that line 288 refers to table 4. However, it is indeed an OR in this case and is referring to the comparison between the studied drug and comparable drugs within the group of participating physicians; it refers to no table at all. We have added a clear referral to the time frame for which this figure was calculated so as to hopefully avoid confusion, as well as changing the subtitle of this section as mentioned in a previous comment.

*9. Throughout, it is not necessary to have sentences like "table X shows that…" Instead, it is clearer just to make the statement and then cite the referenced table at the end, as in (Table 3).*

Thank you for this specific comment on style, we have edited the manuscript as per your suggestion.

*10. Line 335-337. This explanation does not seem adequate. If exposed physicians were already more interested in the study drug in t0, then they would have been matched to higher-prescribing controls in t0. Matching would seem to have accounted for this.*

We agree that the prescription volume is one indicator of a physicians’ interest in a specific drug, which is why we used it as a matching parameter. However, it is possible that certain physicians may have a higher interest in a drug without prescribing it more, for example because they have only recently become aware of it or feel they need to learn more before using it more widely. We were not able to assess such factors and thus believe that it is a limitation that should be mentioned.

*11. In discussion, consider comparing to findings by HE Glass, 2003:* [*https://europepmc.org/article/med/15035835*](https://europepmc.org/article/med/15035835)

Thank you for pointing out this article. We have added it to our discussion as follows: “*Glass studied relative grant amounts from pharmaceutical companies to physicians participating in phase III trials and found no correlation between the relative grant amount and the subsequent prescription behavior of participating physicians, in line with our result that the appropriateness of remuneration for the NIPMS was not associated with the effect on prescription behavior [28].” (lines 415 ff).*

*12. The conclusion that NIPMS should be stopped is overly strong without a much fuller discussion of what the potential benefits and harms of these types of studies are.*

Indeed, we also did not conclude that NIPMS should be stopped completely. However, considering that they have risks that were previously not known (i.e. influencing physician prescribing behavior), and only little benefit as we described in the introduction, we think they should undergo similar scrutiny as clinical trials. We have changed the last sentence to hopefully make this more clear: “*This leads to our conclusion that NIPMS should only be permitted when they are imposed by regulatory authorities or registered with a scientifically sound study design that allows for the collection of essential data.” (lines 433 ff).*

**Reviewer #3**

*[Major Points]
1. This authors' perspective is very unique. The article is worth publication with the condition that the following are described clearly.
(1) Page7, Line128: What is the "Overall prescription volume"? Does it mean that a number of prescriptions per the doctor, per the drug, or per a certain period (e.g. per a month)? And what the aim of using it for control matching.*

We are grateful for the opportunity to clarify. The overall prescription volume refers to the total number of packages of all drugs prescribed by a physician in a certain time frame (t0, t1 or t2). We used this parameter for matching because due to privacy laws we had no identifying information on physicians. We assumed that the overall prescription volume is a marker for the size of the practice and could thus be used to control for this confounder. We added this explanation in the manuscript as follows: *“Prescription data regarding overall prescription volume (i.e. number of packages of all drugs prescribed by a physician) as well as prescriptions of the drug studied in the NIPMS and alternative drugs were acquired for the year before the NIPMS (t0), during the NIPMS (t1) as well as the following year (t2) (see S1 Appendix for definition of “alternative drug”).” (lines 161 ff).*

*(2) Page7, Line133-137: As for Definitions of the "indicators of marketing use", the author just refereed their previous report but it should be described in this paper because these are necessary to interpreter the result accurately. I was not able to access the report on pub-med as probably that is written in German.*

We are happy to offer more detail on the marketing indicators and have added a more detailed description in the S4 Appendix.

*(3) Page9, Line171: What is the purpose of setting two periods of each four months for gathering the physicians' identified data?*

The two periods we initially mentioned in the manuscript were due organizational reasons because some of the data was gathered as part of a previous study as mentioned in the methods section. We have now adjusted the manuscript so as to only mention one time period and avoid confusion, beginning with the start of the first period and ending with the end of the second: *“Data regarding the characteristics of the studies as well as the identifying data on physicians were gathered between Oct 6th 2016 and Jan 9th 2018.” (line 206f).*

*(4) Page10, Line189: What are included in the "revenue generated by these prescriptions"? Is it only remuneration for the INPMS or included sales profits etc.?*

The revenue generated refers only to the prescriptions that were written by the physicians, i.e. how much money statutory health insurance providers had to reimburse due to the prescriptions by the physician in the mentioned time frame. It is completely separate from the remuneration for the NIPMS, which is not described in detail in this study. We have edited the sentence to make this more clear as follows: “*The mean revenue generated from prescriptions of NIPMS- and alternative drugs during this time frame, i.e. the amount that needed to be reimbursed by statutory health insurance providers for prescriptions written by the physicians during t1, was also higher for participating physicians with a mean revenue of 226,713€ (SD: 989,087, median: 32,036€) vs. 153,013€ (SD: 615,225, median: 25,521) for controls” (lines 330 ff).*

*(5) Page8, Table1, 3rd Cullum of Inclusion criteria for NIPMS: Dose the "follow up of one year (t2)" mean one year immediately after the end of the INPMS period? If not, which period has acquired?*

It indeed refers to the year immediately after the end of the NIPMS, as described in the methods section (line 164). We have now added this information to table 1 as well.

*(6) Page8, Table1, 7th Cullum of Inclusion criteria for NIPMS: Author have set the "approved at least 6 months before the beginning of the NIPMS" but 12 months were required to be satisfied one year data before INPMS (t0).*

This is indeed true. For some NIPMS, there was only data for 6 months before the begin of an NIPMS. We chose this time frame as a compromise between getting enough data to be representative of the physicians’ prescribing behavior and including NIPMS studying novel drugs.

*(7) Page13, Table2: There are several NIPMS whose duration is over one year. What rules were applied to extract one-year prescription data from one-year or more data?*

We are grateful for the opportunity to clarify this point. T1 is indeed not the same length for each NIPMS. In the conditional Poisson regression model used to analyze the association of group membership (NIPMS vs. control) with prescription behavior, the NIPMS duration was taken into account when evaluating the absolute number of prescribed packages. Implicitly, the model analyzes the number of prescriptions per unit of time. To account for possible overdispersion, a Quasi-Poisson approach was chosen. This allows for different uncertainties associated with different NIPMS. To clarify, the manuscript has been adjusted slightly: *“To account for possible overdispersion, a Quasi-Poisson approach was chosen. The log number of days of the NIPMS were used as offset.” (lines 260 ff).*

*(8) S1 Appendix. Selection of alternative drugs: How has the author identified alternative drugs when there was more than one drug meets the ATC-code criteria? If specified alternative drugs for each NIPMS were stated in S2 Table Detailed information on characteristics of NIPMS, it would be helpful.*

For most drugs, we selected more than one alternative drug (while for some, there was no alternative according to our criteria). Because there are too many alternative drugs to add them to the S2 table, we added a new table with the alternative drugs (S6 table).

*(9) S2 Appendix. Matching Methods: What the aim of taking into account the "similar defined daily dose (DDD)-prescription of the studied drug" for identifying controls? In my understanding it depends on drugs, not on physicians.*

It is true that the defined daily dose of a certain drug is defined for that drug and has nothing to do with the physician. What we were trying to say here is that we used the number of prescribed DDD of the studied drug by a certain physician as a matching criterion to ensure that participating physicians and controls prescribed similar amounts of the studied drug previous to the begin of the NIPMS. The appendix has been edited to make this more clear as follows: *“For every LANR (a unique ID number permanently assigned to each physician) belonging to a physician taking part in an NIPMS, two control-LANR were selected with a similar prescription behavior during t0 (specifically, overall number of prescribed packages, similarnumber of prescribed defined daily doses (DDD)-prescriptions of the studied drug and number of DDD of the comparable drugs) in t0 based on their Mahalanobis distance via R optmatch package (Version: 0.9-8, R environment for statistical computing version 3.4.3).”* (S2 Appendix)

*2. In the discussion, the bellow points should be considered.
(1) Table 3 shows that even the control has a higher number of packages and DDD on t1 than t0 and t2. What could explain this trend most reasonably?*

We have now clarified in the manuscript that t1 is of a different duration than t0 and t2 due to it being the duration of NIPMS, most of which were longer than one year. This explains the higher number of prescriptions during this time frame. We have added a legend to the table to clarify: “*Note that t1 is not the same duration as t0 and t2;* *number of prescriptions may be compared between groups during the same time frame, but not between time frames.”* We thus do not believe it warrants further consideration in the discussion.

*(2) Table 4 shows that there is a statistical difference in t0, before INPMS. It seems to indicate that physicians participating in a NIPMS already had a preference for new drugs (studied drugs) over existing drugs (alternative drugs), regardless of experience on NIPMS.*

There was indeed a difference between the groups at t0, which we have assessed in response to another reviewers comment as well. There was an average relative difference of 8.29% for number of packages and 10.13% for number of DDD of the studied drug between cases and controls during time period t0. To account for these remaining differences in prescription volume despite matching, Poisson regression models for time period t1 adjusted for the number of NIPMS drugs prescriptions during t0, and for the total number of prescriptions during t1. We would therefore argue that the observed higher relative prescription volume at t1 of NIPMS participants cannot be attributed to self-selection effects with respect to NIPMS participation before t1 in the sense of confounding by indication as the reviewer suggests. Still, we mention in our limitations that there is the rather unlikely possibility that physicians already interested in a specific drug will be more likely to participated in an NIPMS. This seems unlikely because pharmaceutical companies are more likely to target physicians that are not already prescribing the drug (cf. lines 395 ff)

*(3) In S2 Table, there is a large variety of "Duration approval date until begin (mo)" on NIPMS. In general, a situation of using drugs is affected by the time after approval. How did it affect the results of this study?*

One of the marketing indicators was the duration of approval date until begin of the NIPMS, where we considered drugs that had been on the market for over 10 years (trademark drugs) or 5 years (generics) to be too old to warrant an NIPMS because no relevant new information about the drug is to be expected after this time. We assessed whether this marketing indicator was associated with the prescription behavior but found no significant association (see tables 6 and 7).

*(4) In S2 Table, No.1-6 NIPMS have less than 10 physicians. Is there any possibility that few physicians influence the results?*

This is an interesting question. Because our results are weighted according to the number of matching groups in the NIPMS, it is unlikely that these smaller NIPMS affected our results. To study whether NIPMS that include fewer physicians have a different effect on prescription behavior than NIPMS with more physicians, we would need a larger sample size of such small NIPMS.

*[Minor Point]
3. Page6, Line99: there is "Error! Bookmark not defined."*

We have checked the document and errors such as these should no longer occur.

**Reviewer #4**

*I commend the authors in examining the association of the effect of a potential marketing device disguised as a research study with drug prescription.  The retrospective study was based on a fairly large number of physicians of nearly 7000 using what appears to a national claims database that found participation in the NIPMS was associated with higher rates of prescription of the studied medication in the periods during the participation in the NIPMS and the year after.*

We are grateful for this positive assessment of our study.

*Some specific questions and issues to clarify:

1) In the introduction section- Participation in NIPMS.  How do physicians actually "participate" in NIPMS?  How are physicians typically "selected" to participate? This seem to imply the physicians participate in the design/analysis of the study as opposed to something more similar to be detailed (marketed) about the drug.  What are the participants suppose to do and actually do?*

Participation in an NIPMS by a physician usually means that the physician selects patients that are prescribed a specific drug for the enrolment in an NIPMS and is in turn remunerated for the effort of selecting the patient and gathering data. We have added a more precise description of participation in the introduction to make this more clear: “*Physicians’ participation in an NIPMS usually consists of enrolling patients that are prescribed a certain drug in an NIPMS and gathering data on the enrolled patients regarding parameters such as adverse events or efficacy. For the enrolment of each patient, physicians are remunerated with a certain fee” (line 122 ff)*. The pharmaceutical company that sponsors the study and its pharmaceutical representatives select the physicians for participation. According to a German training textbook on pharmaceutical marketing, it is recommended to select physicians for NIPMS who have not yet prescribed the medication as well as key opinion leaders ([1], p. 319). We have already mentioned this aspect in the discussion (lines 395 ff)*.*

[1.]	Trilling T. Pharmamarketing. Berlin, Heidelberg: Springer Berlin Heidelberg; 2015 [cited 2017 Nov 1]. Available from: http://link.springer.com/10.1007/978-3-642-40701-7

*2) In the methods section- National Association of Statutory Health Insurance Funds.  Readers may not be familiar with this.  Is this the name of the national claims database or prescription database?   Or the name of agency?  I assume this database includes all physician prescription database or only containing a subset of physicians or prescriptions?  It seems the database only provide the prescription information for physicians in private practice.  What is the implication of this?  Are most physicians in private practice or work in the hospital?  Is this referring to prescriptions in out-patient versus in-patient settings?*

The National Association of Statutory Health Insurance Funds is the central lobby of the statutory health insurance funds and long-term care insurance funds. It has a wide range of tasks and responsibilities that are not relevant to this study. Relevant to the study, it tracks prescriptions doctors write that are covered by statutory health insurance. In Germany, in 2018, ca. 87% of citizens were insured in statutory health insurance so that most prescriptions are tracked in this database. However, prescriptions are only tracked for physicians working in an out-patient setting in private practice. In Germany, most treatments in an out-patient setting are done by such private practices rather than hospital-associated clinics, which are usually reserved for complex cases. We have added a reference to the number of people insured by a statutory health provider to clarify that our study does not exclude a relevant proportion of patients: “*Prescription data were acquired using data from the GKV-Arzneimittelschnellinformation (GAmSi)-project, which consists of data reported to the National Association of Statutory Health Insurance Funds by pharmacies regarding filled prescriptions for patients with statutory health insurance (in 2018, 87% of German citizens were insured by one of the statutory health insurance providers[19])”(lines 212 ff)*. We don’t believe the manuscript would benefit from more detail on the German health care system because this information is not directly relevant to the study.

*3) In the methods section- Study design on matching to controls.  It is unclear to me (or at least that I can easily decipher) after reading through the methods section what criteria that authors used to match the participants in NIPMS and control.  Matched on overall prescription volume at T0?  What are included in the prescription volume?  Could the patient's specialty impact the prescription volume?  Matched to DDD and packages?  Can the number of years out of training/medical school be found? Table 3- what is the prescription rate normalized to?*

The S2 Appendix contains more information on matching methods, and we have revised this part of the manuscript in reply to another reviewer’s comment and clarify. To summarize, overall prescription volume includes the number of packages of all drugs prescribed by the physician, which was used as an indicator for the size of the practice. Number of prescribed DDD of the studied drug in the NIPMS as well as comparable drugs were also used because we assumed that this would be an indicator of the specialty of the physician, which can of course impact the prescription volume, as the reviewer suggests. Unfortunately, we had no information on the number of years out of training or medical school. The absolute prescription numbers in table 3 were weighted by the number of matching groups in the NIPMS, as mentioned in the legend.

*4) In the Methods section- NIPMS/ Table 2.  The drugs studied is a very heterogenous class of drugs.  Some have alternative equivalent.  Some not as much (i.e. ivabradine).  Some are much more prevalent/commonly used.  In the analysis consider whether categories of class of drugs (cardiac, oncologic, ENT/primary care) affect the association of participation of NIPMS and prescription rate.*

This is a very good point. However, in our sample, we did not have enough different NIPMS and enough matching groups in those NIPMS to allow for meaningful comparisons between drug classes.

*5) Analysis section of outcome measures- Have authors considered measuring "differences in difference" in the prescribing rates of the participants and controls in between the periods T0, T1, and T2?*

Rates (counts per unit of time) are typically modeled using multiplicative relationships since the relevance of observed absolute differences changes with the corresponding absolute level of the counts. Since a “differences in differences” approach would lose the information on absolute level, we did not consider this approach.
