# Supplementary material for: Impact of physicians’ participation in non-interventional post-marketing studies on their prescription habits: A retrospective 2-armed cohort study in Germany
Source: PLoS Med. 2020 Jun 26;17(6):e1003151. doi: 10.1371/journal.pmed.1003151 (PMC7319278; doi:10.1371/journal.pmed.1003151)
Supplement: Attachment — Submitted filename: Response_to_reviewers_2.docx [file pmed.1003151.s016.docx]

**Second Response to Reviewers and Editors regarding the manuscript “Effect of physicians’ participation in non-interventional post-marketing studies on their prescription habits: A retrospective two-armed cohort study” (now: “Impact of physicians’ participation in non-interventional, post-marketing studies on their prescription habits: A retrospective two-armed cohort study in Germany”)**

**Requests from Editors:**

*Title – please add a country setting and as below remove ‘effect’. I suggest a change from Effect of physicians’ participation in non-interventional post-marketing studies on their 2 prescription habits: A retrospective two-armed cohort study
To*
*Impact of physicians’ participation in non-interventional, post-marketing studies on their prescription habits: A retrospective two-armed cohort study in Germany*

We are happy to change the title as suggested.

*Data – thank you for clarifying that the data is not publicly available. Please however add the URL /email  or address or contact for those – who like you did – would like to approach the ministry of Health for access to the data.*

We have amended the data availability statement with the following addition: *“To gain access to the data, readers may contact Cora Koch,* *cora.koch@uniklinik-freiburg.de, who will be of help to approach the respective agencies. The data itself is held by the GKV-Spitzenverband, the National Association of Statutory Health Insurance Funds,* [*https://www.gkv-spitzenverband.de/*](https://www.gkv-spitzenverband.de/)*, Reinhardtstrasse 28, 10117 Berlin, Germany. Access to the data is subject to data privacy restrictions.” (line 540ff)*

*Abstract – please remove “Trial Registration: The trial was not registered, as it was not a clinical trial.”
As this is not a trial, please avoid saying "effect" (e.g. in the title; line 47; line 92; line 360 etc) based on this research design. "Impact" is the maximum that would be acceptable, I would think*

We have changed the abstract and removed the word effect from the manuscript as suggested.

*Throughout manuscript -  more careful about claims throughout, for example, you say "changes prescription rates" at lines 66 and 358 (however, all that is shown is that one group of physicians behave differently from a similar group)*

As you have suggested, we have reread the manuscript and worded more carefully in several places.

*Please briefly tell us about the classes of drugs studied in the abstract, e.g., X of 24 studies involved oncology agents, Y cardiovascular etc.*

We have added information about the two largest studied groups as follows: “*Of the 24 NIPMS, the two largest drug groups studied were antineoplastic or immunomodulatory agents (7/24, 29.2%) and agents for the nervous system (4/24, 16,7%).” (line 58f)*.

*Is it possible to say something about the geography of this study – which cities? If too many to mention, maybe just a brief outline of some information – avoiding a list. Just to give a sense of relevance.*

We are not able to add information for cities for the physicians as we were not allowed access to the personal information of the physicians. We assume that they come from all over Germany, as there were no geographical restrictions on the NIPMS we studied or the sample we took in the matching process. However, we cannot say from where exactly they were.

*You mention "patient harm" at line 97; I don't think there is any data on patient harm, is there? If so, I don't think you should imply that harm might result, rather saying, at most, "we have no data on patient benefits or harms".*

Indeed, we don’t have any data on patient harm, but our point rests on the fact that harm *may* result, unlike previously thought, where NIPMS were assumed to be completely non-interventional for patient care. The reason we believe that NIPMS should be regulated more strictly is that we fear they may cause patients harm; thus they should only be allowed if on the other hand, they confer a benefit, such as essential data regarding the studied drug. However, we have worded the sentence slightly differently to avoid suggesting a strong risk of patient harm: *“However, with the possibility that physicians prescribe differently medication due to their participation in an NIPMS, which may or may be detrimental to patientsnot cause patient harm, only NIPMS that are designed to collect essential data should be permitted.” (line 96ff)*

*STROBE checklist – please use sections and paragraphs instead of pages as these change during revisions and formatting, etc.*

We have edited the checklist accordingly.

**Reviewer #1**

*Thanks for the authors detailed response to my review. Their additional explanations are very helpful in helping clarifying some of my understanding, in particular how the authors minimised the effect of confounding for voluntary NIPMS participation by physicians.

In the response to this they have provided good justification on assessing matching criteria on the relative difference between cases and control during time period t0 as well as controlling for differences in the Poisson regression models. I think their approach is pragmatic and it would be strengthen their arguments, if they could detail this in their manuscript

For the SS calculation - this is fine as a justification that they erred on a conservative approach to try to sample larger numbers as they felt a trial results may lead to under-powering their study. 

One final comment is that the results clearly show that there is increase in prescribing behaviour for the therapeutic products being involved in NIPMS, therefore it would most likely make sense that there might decrease rates in therapeutic products for the same indication which patients could be switched off from. Would the authors comment on whether they believe this effect could be observed?*

We are glad that we have addressed most of the reviewer’s concerns.

Regarding the comment on the justification for our matching criteria, we have added one more explanation regarding the matching in the section on bias so as to hopefully strengthen our argument further as follows: *“We assumed the prior prescription behavior regarding the studied drug to be a good indicator of the previous interest of the physicians in the studied drug.” (line 242ff)*

Regarding the final comment, we did indeed see a shift in the number of prescriptions toward the studied drug, indicating that alternative drugs were prescribed less frequently. However, it is not possible to analyze with the data available to us whether this resulted from a switch of patients to a new medication or whether new patients were simply more likely to be treated with the studied drug. While it would indeed be interesting to analyze the shifts in prescription behavior of physicians participating in an NIPMS in more detail, these analyses would broaden the scope of this manuscript too much.

**Reviewer #2**

*Major comments:
1. Lines 186-188. Were the matching criteria (1) number of overall prescriptions in packages and (2) DDD of the studied drug and (3) DDD of alternative drugs? Or, (1) number of overall prescriptions in packages and (2) DDD of (the studied drug + alternative drugs)? Or something else? This should be clearer. Additionally, were both of these criteria (or all three, if that is the case) factored in equally to the matching?*

We are happy to clarify the matching process further. Three criteria were used as mentioned initially by the reviewer: (1) number of overall prescriptions in packages and (2) DDD of the studied drug and (3) DDD of alternative drugs. We have edited the appendix to avoid any confusion as follows: “*Specifically, three criteria were used to assess prescription behavior and factored equally into matching: overall number of prescribed packages, number of prescribed defined daily doses (DDD) of the studied drug and number of DDD of the comparable drugs.” (S2 Appendix)*

*2. Related to #1 above, but also to my prior comment on the initial submission (Reviewer 2, minor recommendation #10), I am still not clear how, in a strictly mathematical/statistical sense, the t0 differences in prescribing presented in table 4 can be present if groups have been matching on t0 prescribing. If matching occurred based on (overall prescriptions) and (studied drug + alternative drugs), then it seems plausible that such a difference could arise if NIPMS physicians had at baseline a greater % of studied drug within the studied drug + alternative drug total. However, if matching used (overall prescriptions) and (studied drug) and (alternative drugs) separately, then it is not clear to me how the differences in table 4 persisted. Especially since it does not appear that the models were adjusted for other potential confounders, besides these prescribing measures?*

We have clarified the matching process further in our previous comment. The effect the reviewer mentions, that the prescription behavior regarding the studied drug is different at t0 results from the fact that three parameters were used for the matching process and an optimal match was based on the best match for all three parameters. Table 4 shows one of these parameters, adjusted for one other parameter, but not for the third (number of prescriptions of alternative drugs). It is thus possible that the physicians were more closely matched for the number of prescriptions of alternative drugs than for the number of prescriptions of the studied drug, which would explain the remaining difference in table 4.

Minor comments:
*1. Line 120. I believe the authors are making a valid point about publication bias here. However, the phrasing of "NIMPS are also published extremely rarely" might be confused by the reader to mean "NIMPS are also conducted extremely rarely." I would suggest rephrasing to make it clear the NIMPS are often conducted but rarely published.*

We have clarified the sentence as follows, so as to avoid misinterpretation by readers: *“NIPMS are also published extremely rarely, even though they are conducted regularly, which makes it even less likely that they will lead to safer prescription practices, even if relevant data is generated [8,10–12].” (line 120ff)*

*2. Line 177-178. As phrased, it sounds as though the authors had to get approval from the individual NIPMS in order to get data on physician prescribing practices, which I do not expect was the intended meaning.*

Indeed, this was not our intended meaning. We have reworded the sentence as follows: *“We considered NIPMS to be eligible for our study when certain criteria were met allowed for an assessment of the prescription practices of the physicians between one year before and one year after the NIPMS.” (line 178ff)*

**Reviewer #3**

The authors dedicated to clarifying the method of the study by revising it but there are still unclear points especially in the results. To help readers understand the manuscript without misinterpretation, I recommend additional revisions.

[Major Points]
*1.      Page 16, Line 307, "7-8% more of the studied drug during the NIPMS and 6-7% more during the year after the NIPMS" The figures have one digit after the decimal point, and how to calculated these percentages from the number of prescriptions in Table 3.*

As mentioned at the end of the sentence, where it refers to table 4, it merely summarizes the results of table 4. There we found relative prescription rates between 1.07 and 1.08 for the time frame of the NIPMS, corresponding to an 7-8% increase in prescriptions. We reworded the sentence to be more clear: *„Relative to physicians not participating in an NIPMS, physicians participating in an NIPMS had a 7-8% higher prescription rate of the studied drug during the NIPMS, and a 6-7% higher prescription rate during the year after the NIPMS had finished, when accounting for their overall prescription volume during the respective timeframe as well as the number of prescriptions of the studied drug before the begin of the NIPMS (see table 4).“*

*2.      Page 18, Line 329, I'm not sure why the author examines only during t1 (NIPMS) not t2 (immediate after NIPMs) time frame for proportions of prescription of the studied drugs compared to alternative drugs. But this fact should be explicitly stated on page 20, line 362 of the discussion section.*

The pre-specified hypotheses with respect to the proportion of prescription of the studied drugs compared to alternative drugs only covered t1. In the interest of parsimony, we did not run a corresponding exploratory analysis for t2 after data became available. This is now made explicit in the methods section: *“This analysis was only carried out for t1 in the interest of parsimony.” (line 271f)* and in the discussion: *“In addition, they were more likely to prescribe the studied drug rather than alternative drugs used for the same indication during the NIPMS.” (line 366f)*

*3.      Page 18, Table 5, The percentage in parentheses do not match the figures calculated by dividing each number of NIPMS by 24. Besides I don't understand what the author intended to explain from "not appropriate: 4 (14.8%); unclear whether appropriate: 6 (22.2%)."*

Thank you for pointing out this very important mistake – the table was based on an earlier data set which included 3 more NIPMS that were belatedly excluded (as described in S2 Figure due to the late marketing authorization of the studied drugs). The method regarding the assessment of appropriateness of the remuneration is described in detail in the appendix as follows: *“The appropriateness was then rated independently by two raters and where the two raters differed, a consensus was reached after a discussion. A remuneration above the mentioned €75 per hour was considered inappropriate (2 pts. on the marketing index). When there was insufficient information to determine appropriateness, the remuneration was rated as insufficiently justified (1 pt. on the marketing index). All other remuneration was considered appropriate (0 pts. on the marketing index).” (S4 Appendix)*

*4.      Page 18, Table 5, The statements of marketing indicators should be described in the same terms as on the S4 Appendix and S2 Table.*

We have adjusted the wording of the tables and the appendix to match; to keep S2 table readable, the heading for the marketing indicator relating to remuneration remains “Appropriateness of remuneration” as “Remuneration is inappropriate or not clearly warranted” is too long and we expect readers to be able to make the connection even with the slightly different wording.

*[Minor Points]
5.      Page 17, Table 3, I understood the periods are different among the t0, t1. If the average period of each time frame is shown in Table 3, it would be easily recognized the difference.*

Thank you for this good idea. We have added the duration as suggested.

*6.      Before publication the author should look though details of the manuscript since there are several typos (i.e. lack of space; page 2, line 58, "NIPMSprescribed," page 16, line 304, "prescriptionsof").*

This seems to be a formatting problem when converting from word to pdf, but we have tried to avoid such typos in the current version.
